# Review of the millipede genus *Eutrichodesmus* Silvestri, 1910, in China, with descriptions of new cavernicolous species (Diplopoda, Polydesmida, Haplodesmidae)

**DOI:** 10.3897/zookeys.505.9862

**Published:** 2015-05-21

**Authors:** Sergei I. Golovatch, Jean-Jacques Geoffroy, Jean-Paul Mauriès, Didier VandenSpiegel

**Affiliations:** 1 Institute for Problems of Ecology and Evolution, Russian Academy of Sciences, Moscow, Russia; 2 Muséum national d’Histoire naturelle, Département Ecologie & Gestion de la Biodiversité, UMR 7204 CESCO CNRS-MNHN-UPMC, Brunoy, France; 3 Muséum national d’Histoire naturelle, Département Systématique et Evolution, Section Arthropodes, Paris, France; 4 Musée Royal de l’Afrique centrale, Tervuren, Belgium

**Keywords:** Diplopoda, Haplodesmidae, *Eutrichodesmus*, taxonomy, new species, cave, China

## Abstract

The *Eutrichodesmus* fauna of mainland China, by far the largest genus in the Indo-Australian family Haplodesmidae, is reviewed and shown to encompass 23 species (of a total of 45), all keyed. The following nine new species, all presumed troglobites, are described: *Eutrichodesmus triangularis* **sp. n.**, from Sichuan, *Eutrichodesmus lipsae* **sp. n.**, from Guangxi, *Eutrichodesmus tenuis* **sp. n.**, *Eutrichodesmus trontelji* **sp. n.**, *Eutrichodesmus latellai* **sp. n.**, *Eutrichodesmus obliteratus* **sp. n.** and *Eutrichodesmus troglobius* **sp. n.**, all from Guizhou, *Eutrichodesmus sketi* **sp. n.**, from Hunan, and *Eutrichodesmus apicalis* **sp. n.**, from Hubei.

## Introduction

The millipede family Haplodesmidae Cook, 1895, which has only seven component genera basically occurring (except for a few pantropical introductions) in South, East and Southeast Asia, as well as the southwestern Pacific region and Australia, has recently been reviewed (Golovatch et al. 2009a, 2009b, 2010, Golovatch and VandenSpiegel 2014). The most speciose genus is *Eutrichodesmus* Silvestri, 1910, which contains 36 described species and ranges from southern Japan in the north, through Taiwan, southern China and Indochina, to Vanuatu, Melanesia in the south. Mainland China alone supports the following 14 species, mostly from caves (Zhang and Wang 1993, Zhang 1995a, 1995b, Golovatch et al. 2009a, 2009b, 2010; Makhan 2010, Liu and Tian 2013):

*Eutrichodesmus anisodentus* (Zhang, 1995), from Mt. Wuyi, Fujian Prov. (Zhang 1995b, Golovatch et al. 2010);

*Eutrichodesmus arcicollaris* Zhang in Zhang & Wang, 1993, from Cave Huayu Dong, Hekou County, Yunnan Prov. (Zhang and Wang 1993, Golovatch et al. 2009a, 2009b);

*Eutrichodesmus digitatus* Liu & Tian, 2013, from Cave Mi Dong, Jintan Town, Qingyuan City, Guangdong Prov. (Liu and Tian 2013);

*Eutrichodesmus distinctus* Golovatch, Geoffroy, Mauriès & VandenSpiegel, 2009, from Cave 4, Bapen, Fushui County, Guangxi Prov. (Golovatch et al. 2009b);

*Eutrichodesmus dorsiangulatus* (Zhang in Zhang & Wang, 1993), from Cave Baoniujiao Dong, Mengla County, Yunnan Prov. (Zhang and Wang 1993, Golovatch et al. 2009a, 2009b);

*Eutrichodesmus incisus* Golovatch, Geoffroy, Mauriès & VandenSpiegel, 2009, from caves near Hong Lin, Qianxi County, Guizhou Prov. (Golovatch et al. 2009a);

*Eutrichodesmus latus* Golovatch, Geoffroy, Mauriès & VandenSpiegel, 2009, from caves in Yachang Nature Reserve, Guangxi Prov. (Golovatch et al. 2009a);

*Eutrichodesmus monodentus* (Zhang in Zhang & Wang, 1993), from Cave Caiyun Dong, Mengla County, Yunnan Prov. (Zhang and Wang 1993, Golovatch et al. 2009a, 2009b);

*Eutrichodesmus pectinatidentis* (Zhang, 1995), from Mt Tianmu, Lin’an County, Zhejiang Prov. (Zhang 1995a, Golovatch et al. 2010);

*Eutrichodesmus planatus* Liu & Tian, 2013, from Cave Zhenzhuyan Dong, Liujia Town, Hechi City, Guangxi Prov. (Liu and Tian 2013);

*Eutrichodesmus similis* Golovatch, Geoffroy, Mauriès & VandenSpiegel, 2009, from several caves in Mulun Nature Reserve, Huanjiang County, Guangxi Prov. (Golovatch et al. 2009a, Liu and Tian 2013);

*Eutrichodesmus simplex* Liu & Tian, 2013, from Cave Taoyuan Dong, Fenyi County, Jiangxi Prov. (Liu and Tian 2013);

*Eutrichodesmus soesilae* Makhan, 2010, from Mt. Jinyun, Beibei, Chongqing Municipality (Makhan 2010, Golovatch et al. 2010);

*Eutrichodesmus spinatus* Liu & Tian, 2013, from Sidu Caves, Sidu Town, Hunan Prov. (Liu and Tian 2013).

The present paper puts on record another nine new species of *Eutrichodesmus* from Chinese caves, being concluded by a key to all 23 species of the genus currently known to occur in mainland China.

### Abbreviations used

MNHN Muséum national d’Histoire naturelle, Paris, France

SEM Scanning electron microscopy

## Material and methods

The material serving as the basis for the present contribution derives from subterranean collections made in China by Josiane Lips (Villeurbanne, France), Leonardo Latella and Daniele Avesani (both from the Museo Civico di Storia naturale, Verona, Italy), as well as Boris Sket, Peter Trontelj and their collaborators (all from the University of Ljubljana, Slovenia). All material, including the holotypes, has been deposited in MNHN. The term “doratodesmoid” is used hereafter only in its vernacular meaning, in order to concisely characterize a body shape, i.e. capable or nearly capable of volvation.

SEM micrographs were taken using a JEOL JSM-6480LV scanning electron microscope.

After examination, SEM material was removed from stubs and returned to alcohol, all such samples being kept at MNHN.

## Systematics

### Eutrichodesmus triangularis

Taxon classificationAnimaliaPolydesmidaHaplodesmidae

Golovatch, Geoffroy, Mauriès & VandenSpiegel sp. n.

http://zoobank.org/642BEA4E-D3AA-49FE-B829-8DEA0478E2B4

Figs 1 , 2

#### Type material.

Holotype ♂ (MNHN JC 367), China, Sichuan Prov., Beichuan County, Cave Yan Dong, 18.VIII.2004, leg. J. Lips (No. 1583).

#### Name.

To emphasize the prominent, triangular, distofemoral process (**dp**) of the gonopod; adjective.

#### Diagnosis.

Differs from congeners by the prominent, triangular, distofemoral process of the gonopod (see also Key below).

#### Description.

**Figure 1.**
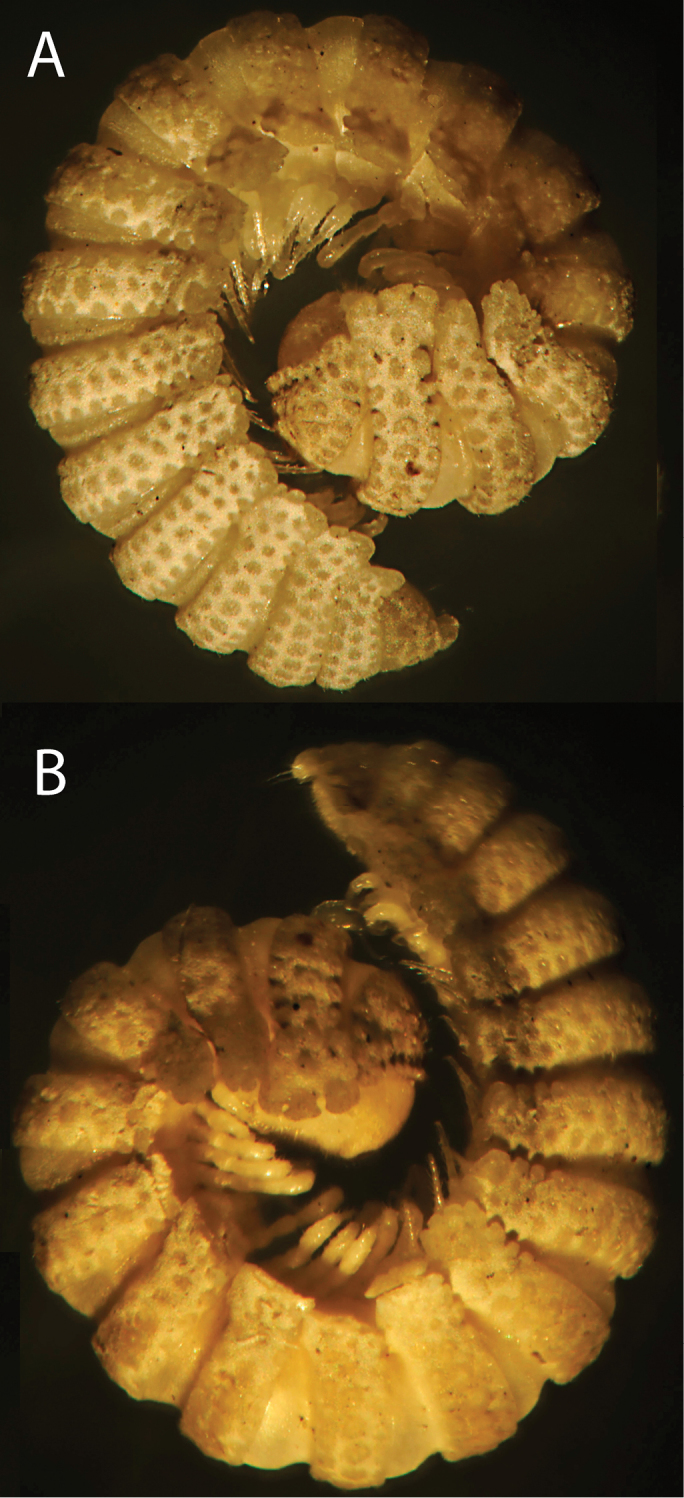
*Eutrichodesmus triangularis* sp. n., ♂ holotype; **A, B** habitus, sublateral and lateral views, respectively. Pictures by A. Kirejtshuk, not taken to scale.

Length ca 7.0 mm, width 0.9 and 1.5 mm on midbody pro- and metazonae, respectively. Coloration uniformly very light brown with pallid antennae, clypeolabral region, prozonae, venter, legs and metatergal tuberculations (Fig. 1).

Body with 19 segments (♂) (Fig. 1), conglobation pattern typical of “doratodesmoids”, volvation apparently being complete because of strongly declivous and relatively narrow paraterga. Tegument dull, meta- and paraterga with a cerategument layer. Antennae short and clavate. Head with a paramedian pair of small, but distinct, rounded tubercles above antennal sockets. Collum not covering the head from above, fore margin slightly elevated, with 4-5 transverse rows of flat tuberculations, first two and caudalmost rows being regular (Fig. 1). Metaterga behind collum with three transverse, rather irregular, mixostictic (= not regularly longitudinal) rows of similarly flat, rounded, obviously setigerous tuberculations extending onto paraterga, usually about 11-12+11-12 per row (Fig. 1); limbus microcrenulate. Paraterga with evident shoulders anteriorly, strongly declivous, directed ventrolaterad at about 45° to subvertical sides above paraterga, broad, tips about level with venter, distinctly trilobate laterally, without anterolaterals, but with 2-3 rounded caudolaterals (Fig. 1). Paraterga 2 rather strongly enlarged, directed ventrolaterad, lateral margin especially deeply trilobate, caudal margin with a row of lobules extending across dorsum, both schism and hyposchism small; paraterga 3 and 4 slightly shorter than others. Pore formula normal, ozopores indistinct, located dorsally between middle and caudolateral lobulations. Pleurotergal carinae wanting. Epiproct fully exposed in dorsal view, rather strongly flattened, dorsally also tuberculate, with several incisions at lateral edge, directed ventrocaudad, with the usual four cones just below tip (Fig. 1). Hypoproct subtrapeziform.

Sterna usually with a deep and narrow depression between coxae. Legs short, crassate except for slender tarsi, about as long as body height.

**Figure 2.**
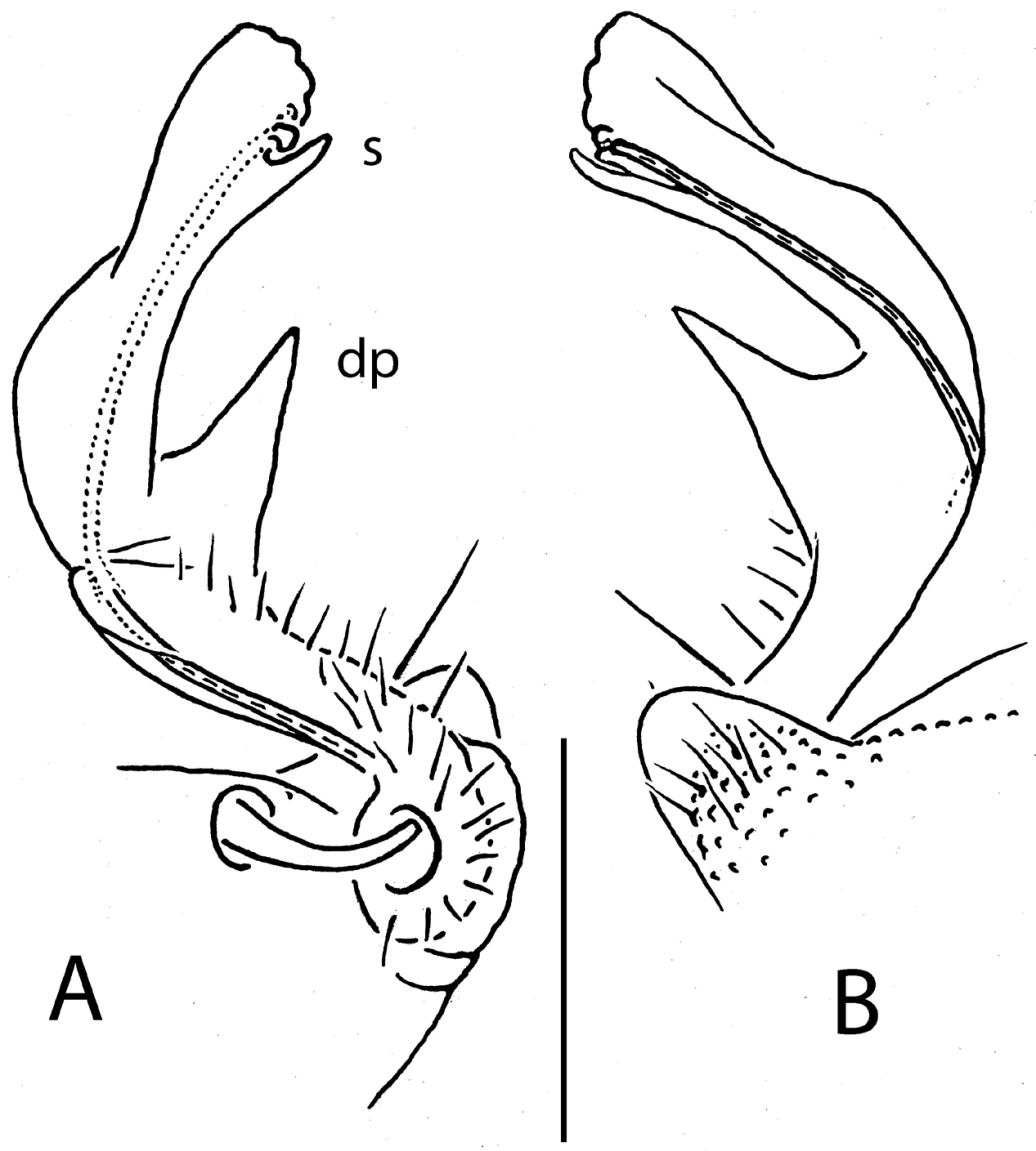
*Eutrichodesmus triangularis* sp. n., ♂ holotype; **A, B** left gonopod, mesal and lateral views, respectively. Scale bar: 0.2 mm. Designations in text.

Gonopods (Fig. 2) simple. Coxae subquadrate, large, microtuberculate and abundantly setose ventrolaterally, with a conspicuous round lobe caudolaterally. Telopodite considerably longer than coxite, but not too slender, subfalcate, distinctly curved ventrad, setose not only in its basal half, including mesal face at base of a prominent, triangular, acuminate, distofemoral process (**dp**), the latter situated at about midway along telopodite, more distally with a lobe-shaped, rounded, distad slightly enlarged acropodite showing a short, distoventral, subapical spine (**s**); seminal groove terminating subapically, devoid of a hairpad.

#### Remarks.

The presence of only 19 body segments is rare in *Eutrichodesmus*, but generally quite common in Haplodesmidae (Golovatch et al. 2009a). Among congeners, the above new species seems to share this feature only with *Eutrichodesmus asteroides* Golovatch, Geoffroy, Mauriès & VandenSpiegel, 2009, from a cave in Vietnam (Golovatch et al. 2009b).

More information on the location of the cave can be found at http://www.groupe-speleo-vulcain.com/explorations/expeditions-a-letranger/

### Eutrichodesmus lipsae

Taxon classificationAnimaliaPolydesmidaHaplodesmidae

Golovatch, Geoffroy, Mauriès & VandenSpiegel sp. n.

http://zoobank.org/36DDFFFF-ACC7-40D2-A056-C1A904393C38

Figs 3 , 4

#### Type material.

Holotype ♂ (MNHN JC 368), China, Guangxi Prov., Guilin County, Grotte des Squelettes, 22.VII.1992, leg. J. Lips (No. B1-2).

Paratypes: 1 ♂ (SEM), 1 juv. (MNHN JC 368), same data, together with holotype.

#### Name.

In honour of Josiane Lips, the collector.

#### Diagnosis.

Differs from congeners by clearly elevated mid-dorsal regions of most metaterga, coupled with a slender, suberect gonopod telopodite which shows a rather narrowly gapped apical pincer (see also Key below).

#### Description.

Length of adults ca 7.0 mm, width 1.0 and 1.95 mm on midbody pro- and metazonae, respectively (♂). Coloration entirely pallid.

All characters as in *Eutrichodesmus triangularis* sp. n., except as follows.

**Figure 3.**
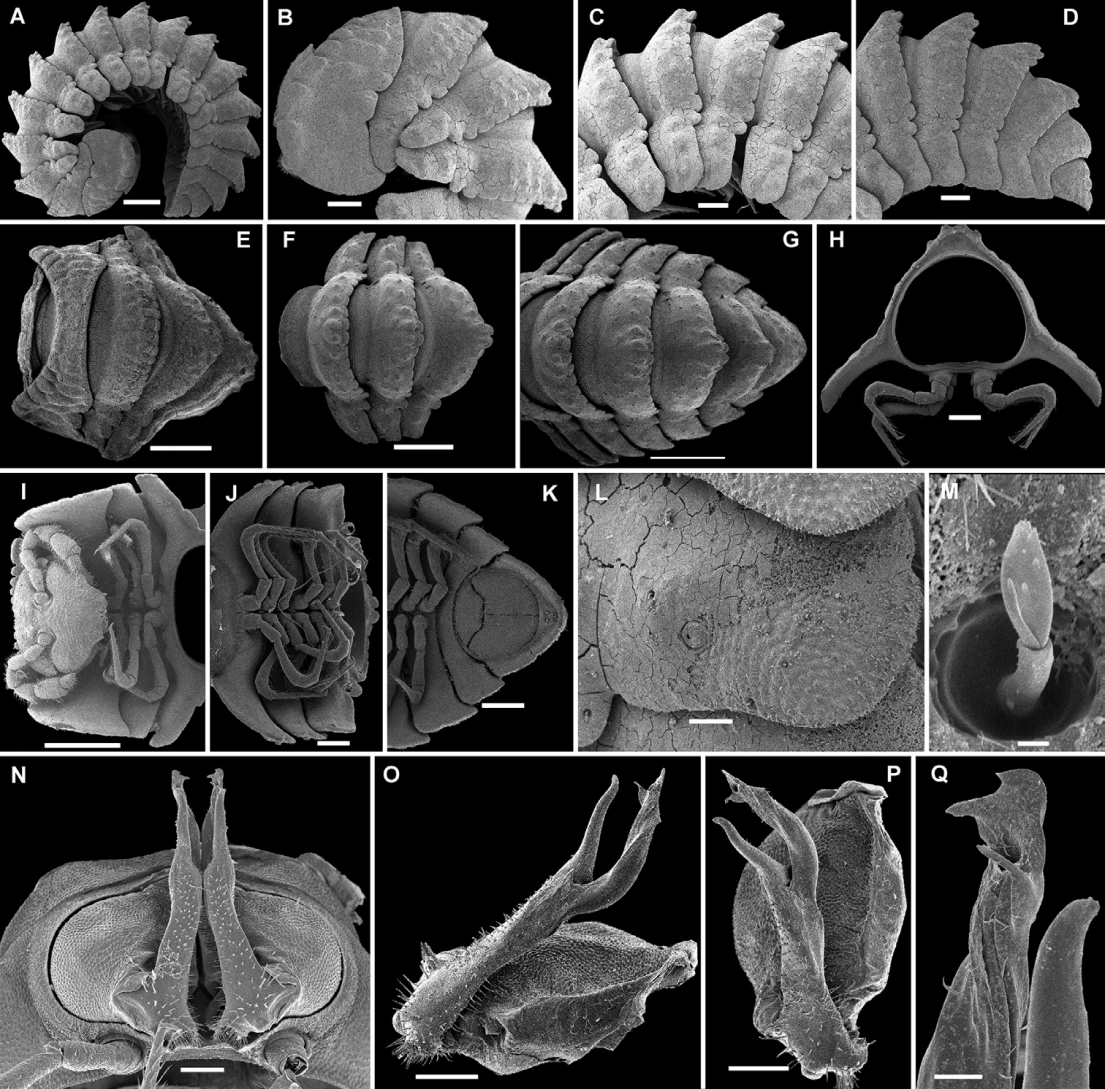
*Eutrichodesmus lipsae* sp. n., ♂ paratype; **A** habitus, lateral view **B, E, I** anterior part of body, lateral, dorsal and ventral views, respectively **C, F, J** midbody segments, lateral, dorsal and ventral views, respectively **D, G, K** posterior part of body, lateral, dorsal and ventral views, respectively **H** cross-section of a midbody segment, caudal view **L** poriferous midbody paratergite, lateral view **M** tergal seta, subdorsal view **N** both gonopods in situ, ventral view **O, P** right gonopod, mesal and ventromesal views, respectively **Q** tip of right gonopod, subventral view. Scale bars: 0.5 mm (**A, E–G, I**), 0.2 mm (**B–D, H, J, K**), 0.1 mm (**N–P**), 0.05 mm (**L**), 0.02 mm (**Q**), 0.002 mm (**M**).

Body with 20 segments (♂) (Fig. 3A), conglobation pattern typical of “doratodesmoids”, volvation apparently being complete because of strongly declivous and relatively narrow paraterga. Antennae short and clavate (Fig. 3I). Collum not covering the head from above, fore margin clearly lobulate, with 4-5 transverse rows of very flat tuberculations/bosses. Metaterga behind collum with three transverse, rather irregular, mixostictic rows of similarly flat, rounded, often obliterate, obviously setigerous bosses extending onto paraterga, usually about 9-10+9-10 per row (Fig. 3A–G); starting with segment 3, middle and caudal rows clearly enlarged and elevated mid-dorsad, increasingly clearly so towards segment 15 or 16 as well (Fig. 3A–G); caudomarginal lobulations evident across dorsum; limbus microcrenulate. Paraterga with evident shoulders anteriorly, strongly declivous, directed ventrolaterad at about 45° to only slightly less strongly declined sides above paraterga, broad, tips lying clearly below level of venter, usually vaguely bilobate laterally, without anterolaterals, but with well-developed rounded caudolaterals at and above base (Fig. 3A–D, L). Paraterga 2 strongly enlarged, directed ventrad (Fig. 3A, B, I), lateral margin broadly rounded, with numerous, very small lobulations, caudal margin with a row of lobules extending across dorsum, both schism and hyposchism small. Tergal setae very short, 2-segmented, apical part phylloid (Fig. 3M). Pore formula normal, ozopores indistinct, located dorsally at about anterior 1/3 of paratergite and well removed from lateral margin (Fig. 3L). Hypoproct subtrapeziform (Fig. 3K).

Sterna usually with a rather deep, narrow depression between coxae (Fig. 3I, J). Legs long and slender, about 1.1-1.2 times as long as body height.

**Figure 4.**
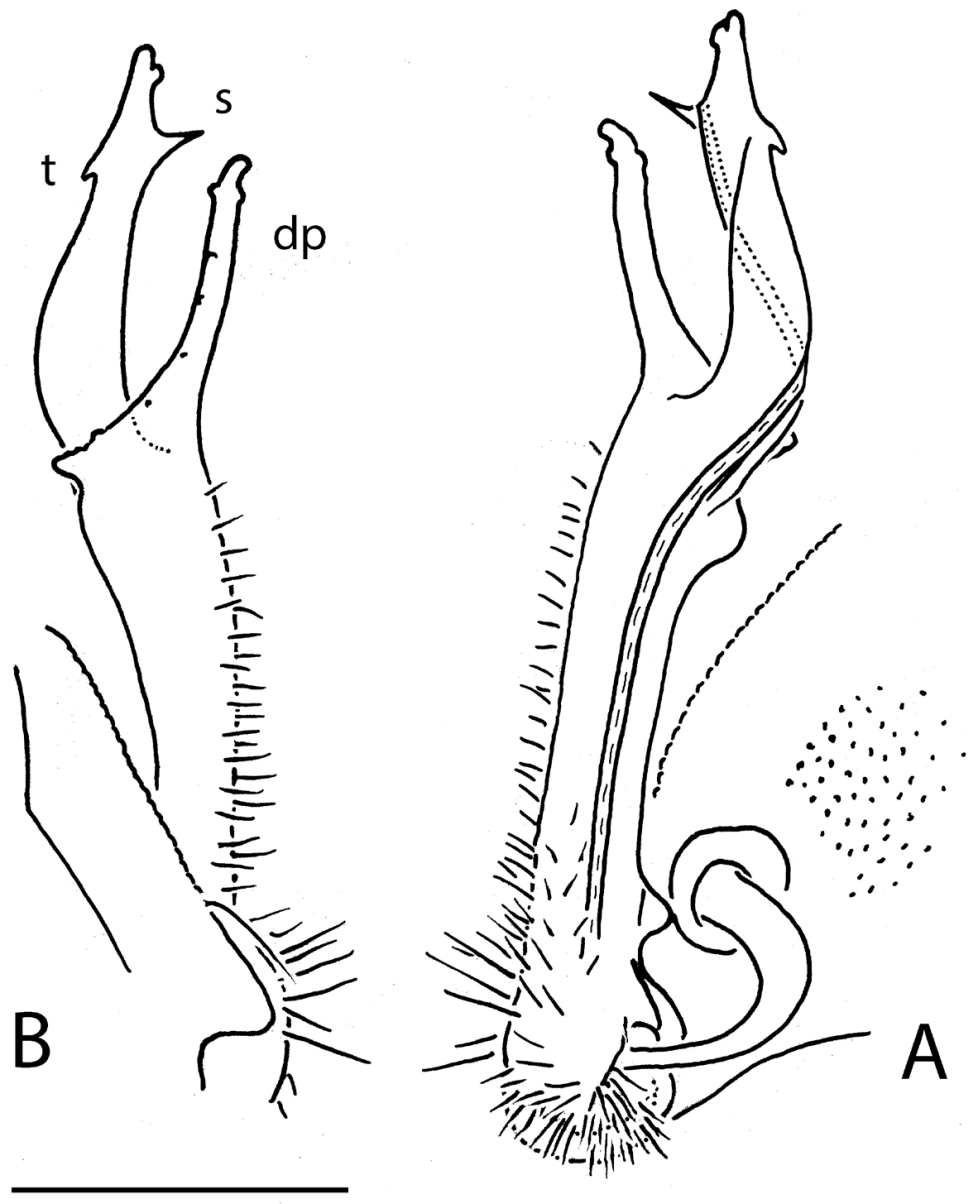
*Eutrichodesmus lipsae* sp. n., ♂ holotype; **A, B** right gonopod, mesal and lateral views, respectively. Scale bar: 0.2 mm.

Gonopods (Figs 3N-Q, 4) simple. Coxae subquadrate, large, micropapillate, but not setose, with only a small round lobe caudolaterally. Telopodite considerably longer than coxite, slender, suberect, setose over its basal 2/3 until base of a prominent, finger-shaped, poorly papillate, distofemoral process (**dp**), the latter situated in distal 1/4 of telopodite, more distally with a rather narrow, twisted, subacuminate, slightly longer acropodite forming a rather narrowly gapped pincer together with **dp** and showing a short, distoventral, subapical spine (**s**) and a small, distodorsal, subapical tooth (**t**); seminal groove terminating at base of **s**, devoid of a hairpad.

#### Remark.

More information on the location of the cave can be found at http://www.groupe-speleo-vulcain.com/explorations/expeditions-a-letranger/

### Eutrichodesmus tenuis

Taxon classificationAnimaliaPolydesmidaHaplodesmidae

Golovatch, Geoffroy, Mauriès & VandenSpiegel sp. n.

http://zoobank.org/B7247170-99F8-4A4F-AED5-440344A4E881

Figs 5 , 6

#### Type material.

Holotype ♂ (MNHN JC 369), China, Guizhou Prov., Guanling County, Yong Ning Town, Cave Yun Dong (Cloud Cave), 01.VIII.2005, leg. L. Latella & D. Avesani.

Paratypes: 1 ♀, 2 subadult ♀ (MNHN JC 369), 1 subadult ♀ (SEM), same data, together with holotype.

#### Name.

To emphasize the relatively slender body due to subvertical paraterga; adjective.

#### Diagnosis.

Differs from congeners by the large body size, clearly elevated mid-dorsal regions of most metaterga, coupled with narrow, strongly declivous, subvertical paraterga and a simple, falcate gonopod telopodite carrying a long, spiniform, distofemoral process (see also Key below).

#### Description.

Length of adults ca 14–15 mm, width 1.8 and 2.5 mm (♂ holotype) or 1.6 and 2.1 mm (♀ paratype) on midbody pro- and metazonae, respectively. Coloration entirely pallid, sometimes (♀ paratype) with traces of reddish earth material on metaterga.

All characters as in *Eutrichodesmus triangularis* sp. n., except as follows.

**Figure 5.**
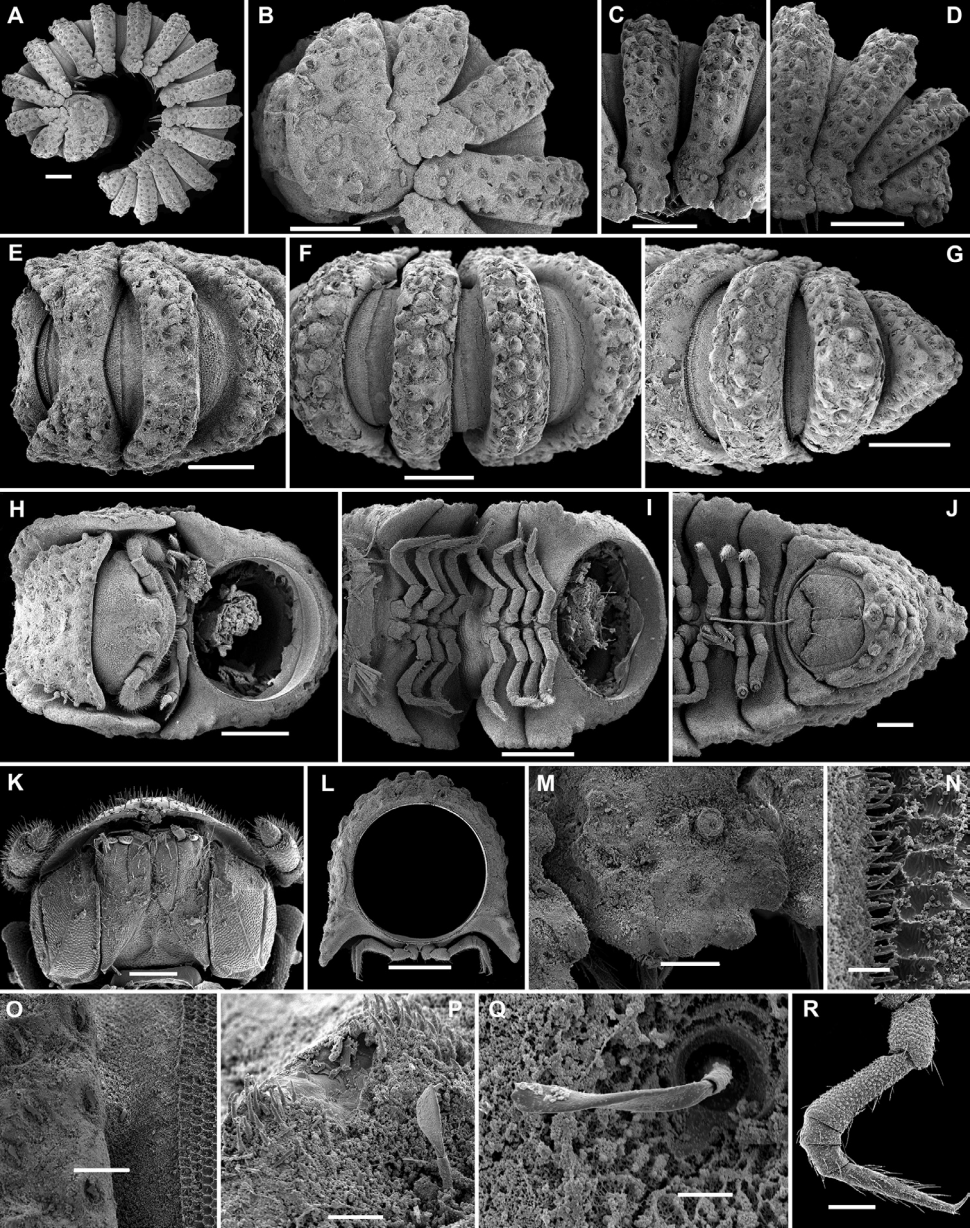
*Eutrichodesmus tenuis* sp. n., subadult ♀ paratype; **A** habitus, lateral view **B, E, H** anterior part of body, lateral, dorsal and ventral views, respectively **C, F, I** midbody segments, lateral, dorsal and ventral views, respectively **D, G, J** posterior part of body, lateral, dorsal and ventral views, respectively **K** head, ventral view **L** cross-section of a midbody segment, caudal view **M** poriferous midbody paratergite, lateral view **N** limbus, lateral view **O, P, Q** tergal setae, various views **R** midbody leg. Scale bars: 0.5 mm (**A–I, L**), 0.2 mm (**J, K**), 0.1 mm (**M, O, R**), 0.02 mm (**N, P**). 0.01 mm (**Q**).

Body with 20 segments (♂, ♀), conglobation pattern typical of “doratodesmoids”, volvation apparently being complete because of particularly strongly declivous and short paraterga. Antennae short and clavate (Fig. 5H, K). Collum not covering the head from above, fore margin clearly lobulate and slightly elevated, with 4-5 transverse rows of small, but evident tuberculations, only frontal- and caudalmost rows being regular. Metaterga behind collum with three transverse, rather irregular, mixostictic rows of similarly evident, rounded, setigerous tuberculations extending onto paraterga, usually about 10-11+10-11 per row (Fig. 5A–G); starting with midbody segments, middle rows clearly enlarged and elevated mid-dorsad, increasingly clearly so towards segment 18 as well (Fig. 5A–D); a few caudomarginal lobulations evident only on paraterga (Fig. 5A–D); limbus microcrenulate (Fig. 5N). Paraterga with evident shoulders anteriorly, very strongly declivous, subvertical, directed ventrolaterad at about 75–80° to even more strongly declined sides above paraterga (Fig. 5L), broad, tips lying clearly below level of venter, usually rather vaguely tri- or quadrilobate laterally, without anterolaterals (Fig. 5A–D, M). Paraterga 2 strongly enlarged, directed ventrad (Fig. 5A, B, E, I), lateral margin broadly rounded, with numerous, very small lobulations, caudal margin with a few lobes located near schism, both schism and hyposchism being small (Fig. 5B). Tergal setae very short, 2-segmented, apical part usually phylloid (Fig. 5P, Q). Pore formula normal, ozopores distinct, located dorsally on small porosteles in posterior 1/3 of paratergite and well removed from lateral margin (Fig. 5A–D, M). Epiproct finger-shaped, densely tuberculate (Fig. 5D, G, J). Hypoproct subtrapeziform (Fig. 5J).

**Figure 6.**
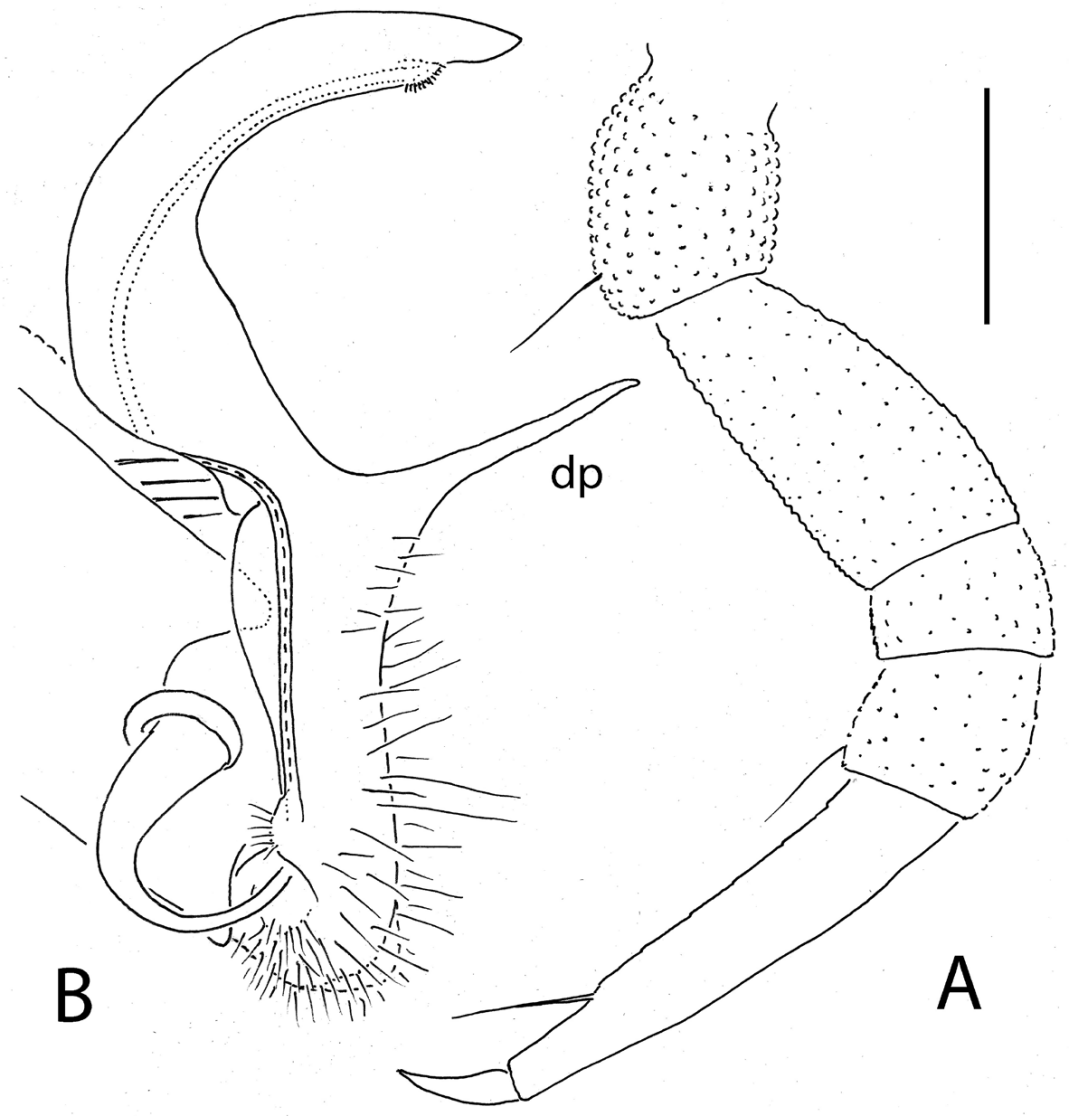
*Eutrichodesmus tenuis* sp. n., ♂ holotype; **A** leg 9, lateral view **B** left gonopod, mesal view. Scale bar: 0.2 mm. Designation in text.

Sterna usually with a rather deep, narrow depression between coxae (Fig. 5I). Legs short and crassate, about half as long as body height (Fig. 5L), all podomeres except tarsi finely micropapillate (Figs 5R, 6A).

Gonopods (Fig. 6B) very simple. Coxae subquadrate, large, micropapillate and rather densely setose on lateral face, with only a small round lobe caudolaterally. Telopodite considerably longer than coxite, suberect, setose over its basal half until base of a prominent, spiniform, simple, distofemoral process (**dp**), the latter situated at about halfway along telopodite, acropodite strongly falcate, twisted, subacuminate, simple, devoid of outgrowths; seminal groove terminating subapically at base of a hairpad.

#### Remark.

More information on this cave and its fauna can be found in Latella and Hu (2008) and in Latella and Zorzin (2008).

### Eutrichodesmus trontelji

Taxon classificationAnimaliaPolydesmidaHaplodesmidae

Golovatch, Geoffroy, Mauriès & VandenSpiegel sp. n.

http://zoobank.org/7016E520-525A-4D21-A47D-83A850193D51

**Figure 7.**
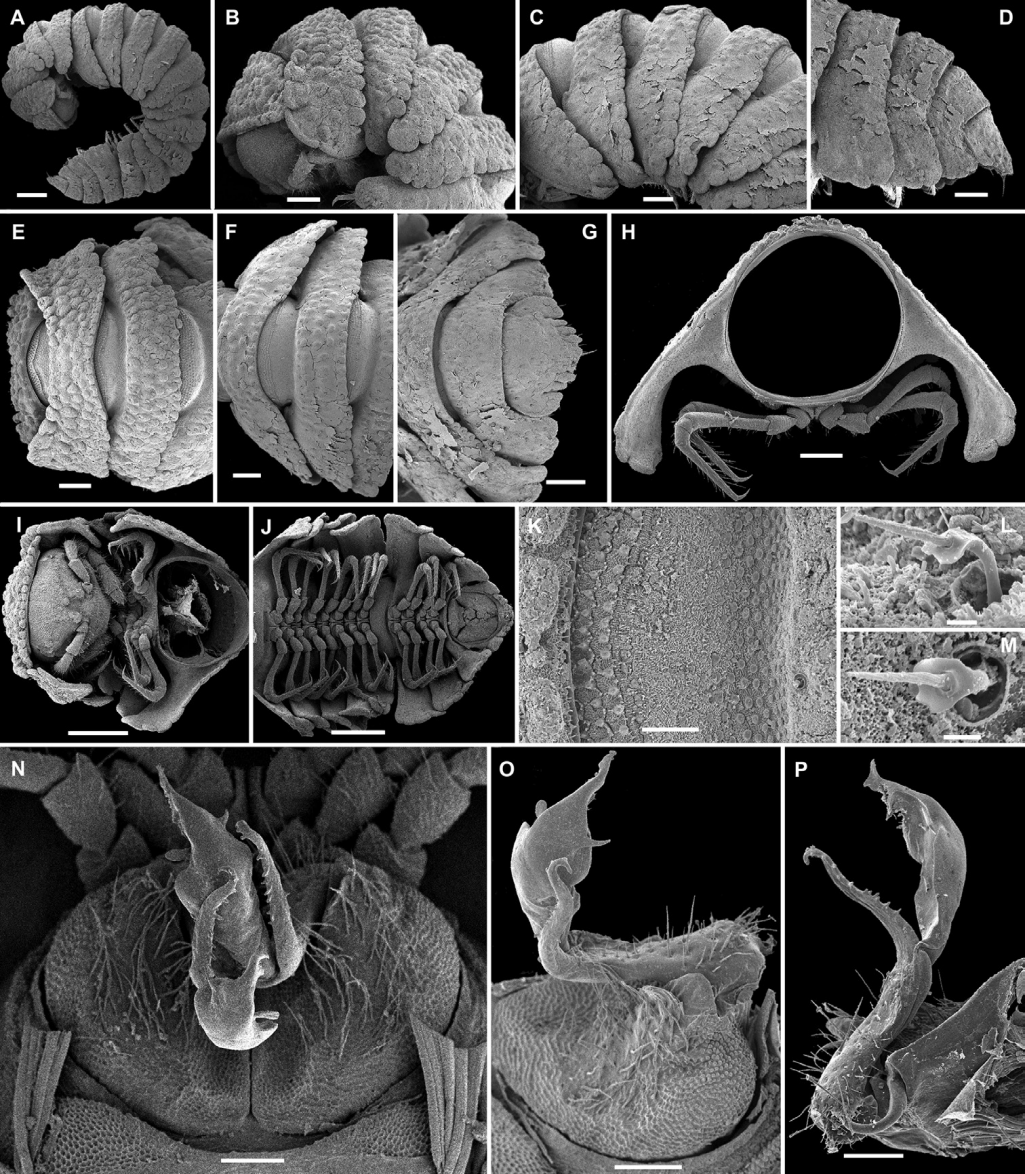
*Eutrichodesmus trontelji* sp. n., ♂ paratype; **A** habitus, lateral view **B, E, I** anterior part of body, lateral, dorsal and ventral views, respectively **C, F** midbody segments, lateral and dorsal views, respectively **D, G, J** posterior part of body, lateral, dorsal and ventral views, respectively **H** cross-section of a midbody segment, caudal view **K** limbus, prozonite texture and tergal setae, dorsal view **L, M** tergal seta, dorsolateral and subdorsal views, respectively **N** both gonopods in situ, ventral view **O, P** right gonopod, lateral and mesal views, respectively. Scale bars: 0.5 mm (**A, I, J**), 0.2 mm (**B–H**), 0.1 mm (**N–P**), 0.05 mm (**K**), 0.005 mm (**L, M**).

**Figure 8.**
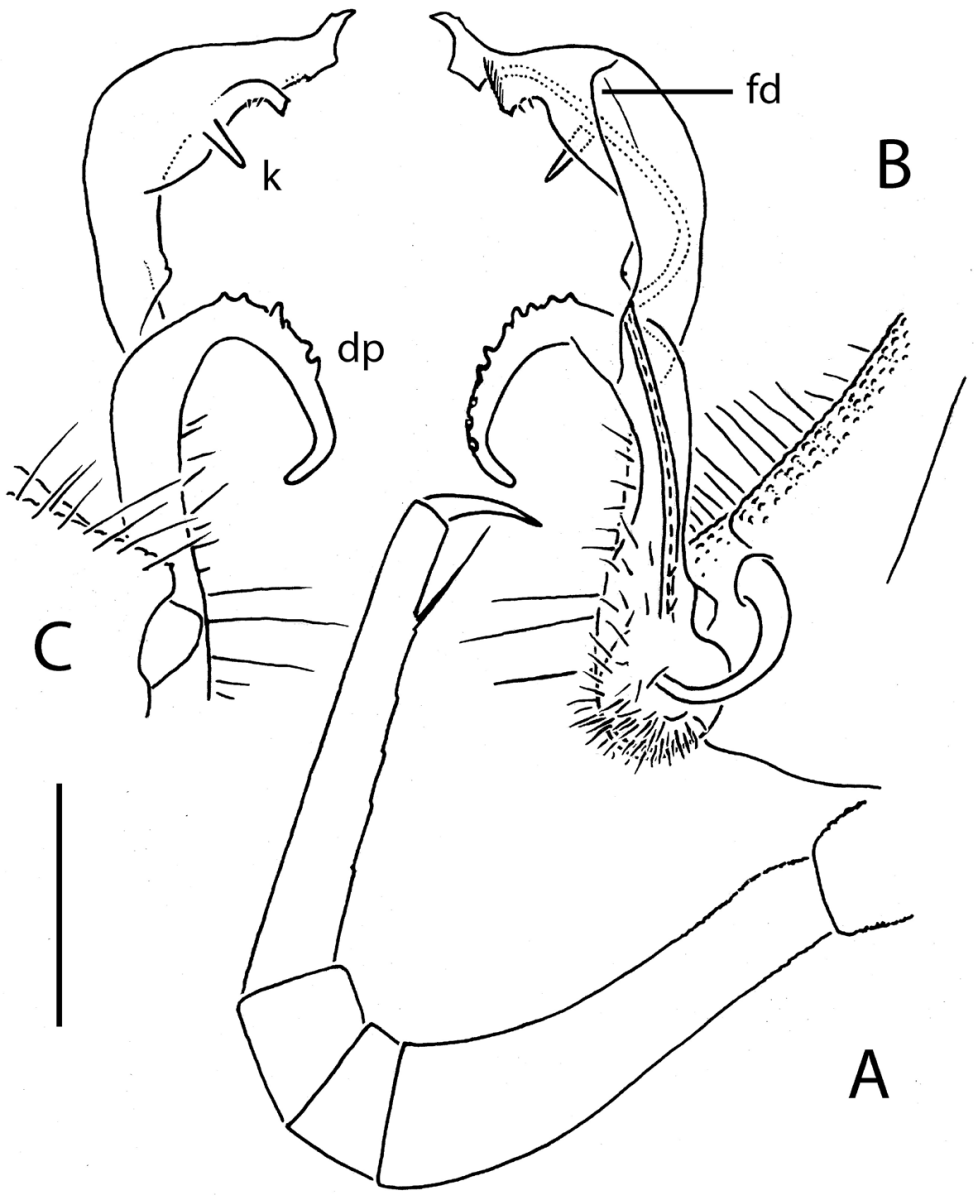
*Eutrichodesmus trontelji* sp. n., ♂ paratype; **A** leg 9, lateral view **B, C** right gonopod, mesal view. Scale bar: 0.2 mm. Designations in text.

**Figure 9.**
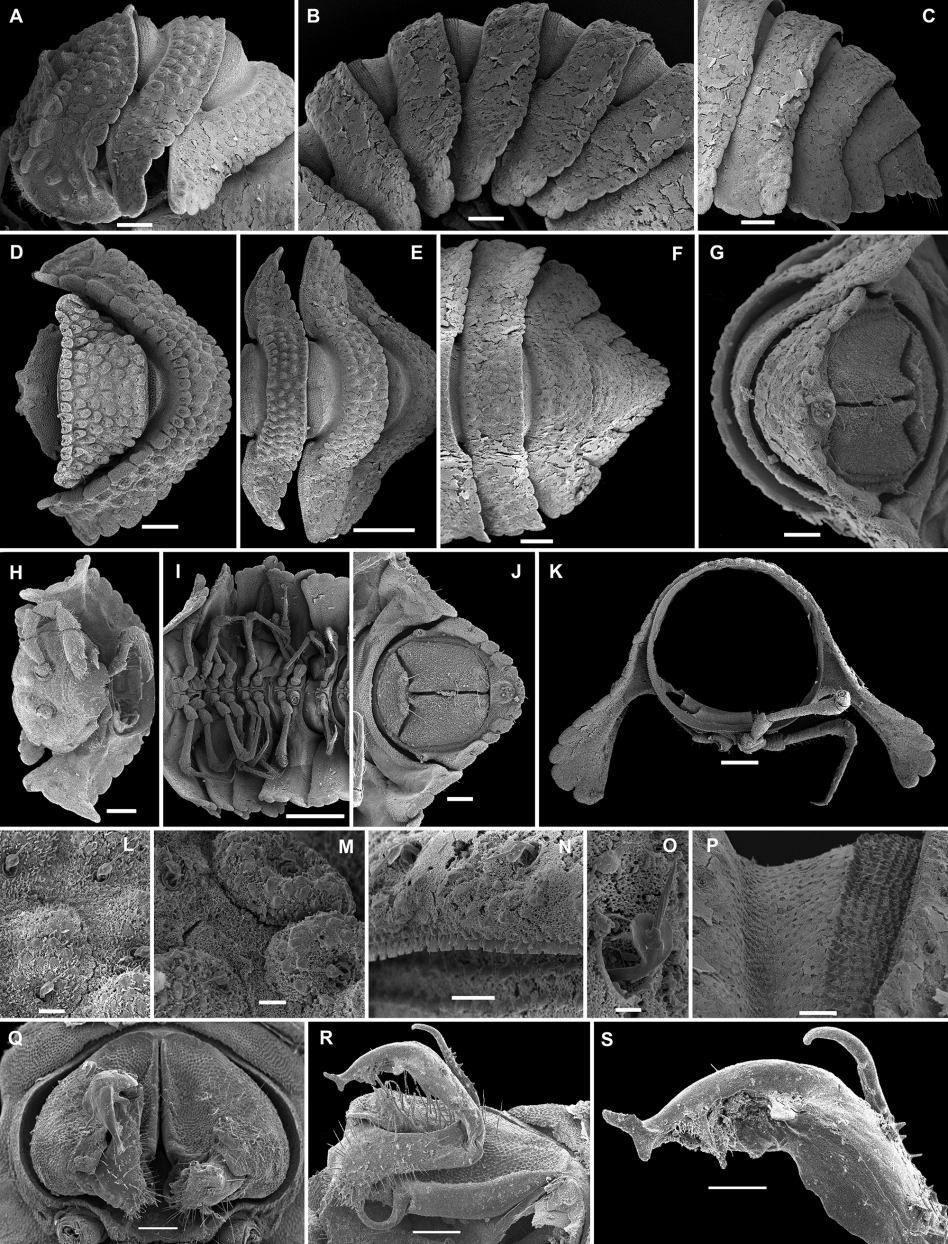
*Eutrichodesmus trontelji* sp. n., ♂ non-type from Shui Jiang Dong; **A, D, H** anterior part of body, lateral, dorsal and ventral views, respectively; **B, E, I**, midbody segments, lateral, dorsal and ventral views, respectively **C, F, G, J** posterior part of body, lateral, dorsal, caudal and ventral views, respectively **K** cross-section of a midbody segment, caudal view **L-P** limbus, prozonite texture and tergal setae, dorsal views **Q** both gonopods in situ, ventral view **R** right gonopod, mesal view **S** distal half of right gonopod, mesal view. Scale bars: 0.5 mm (**E, I**), 0.2 mm (**A–D, F, H, K**), 0.1 mm (**G, J, Q, R**), 0.05 mm (**P, S**), 0.02 mm (**L–N**), 0.005 mm (**O**).

**Figure 10.**
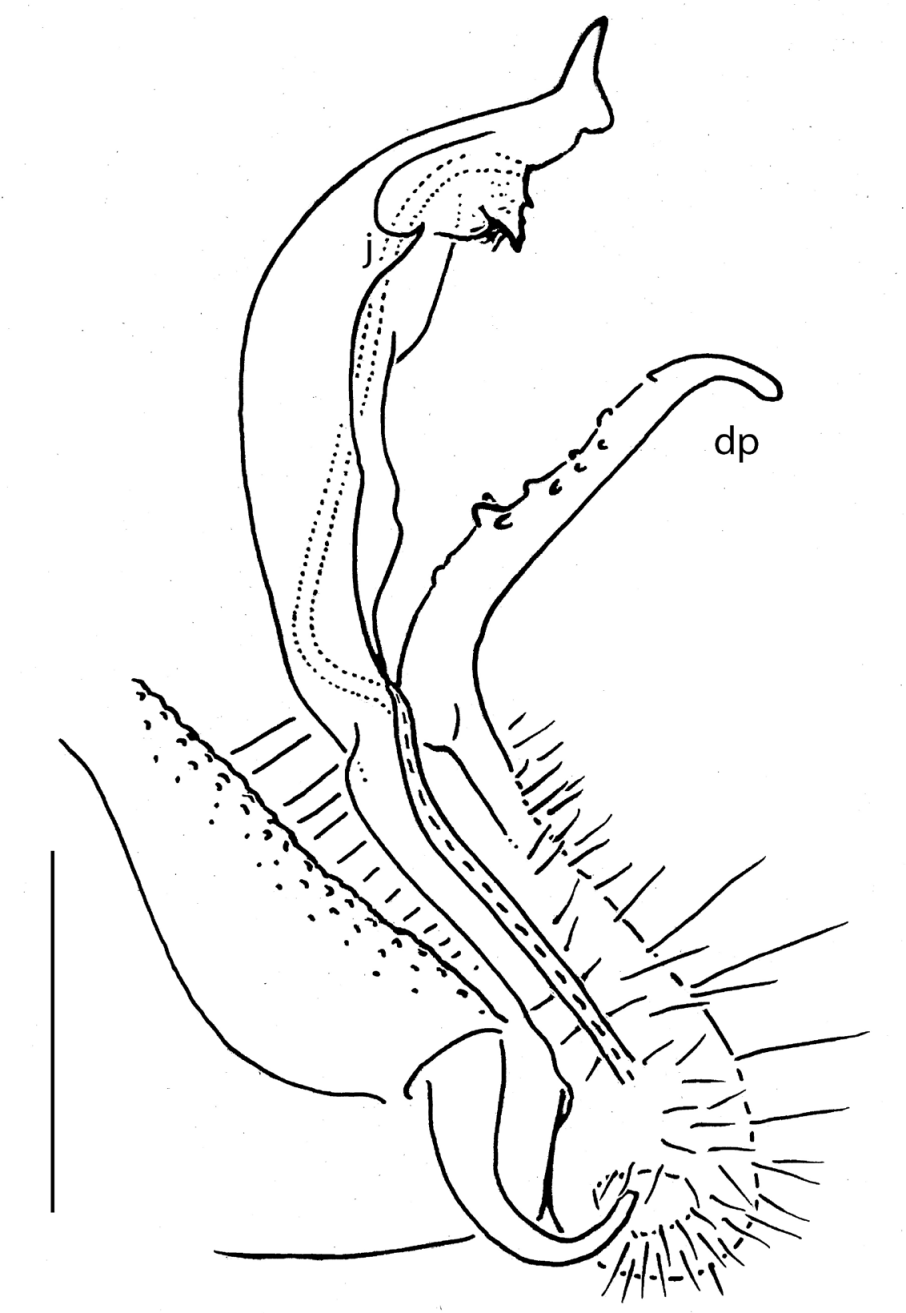
*Eutrichodesmus trontelji* sp. n., ♂ non-type from Shui Jiang Dong, left gonopod, mesal view. Scale bar: 0.2 mm. Designations in text.

**Figure 11.**
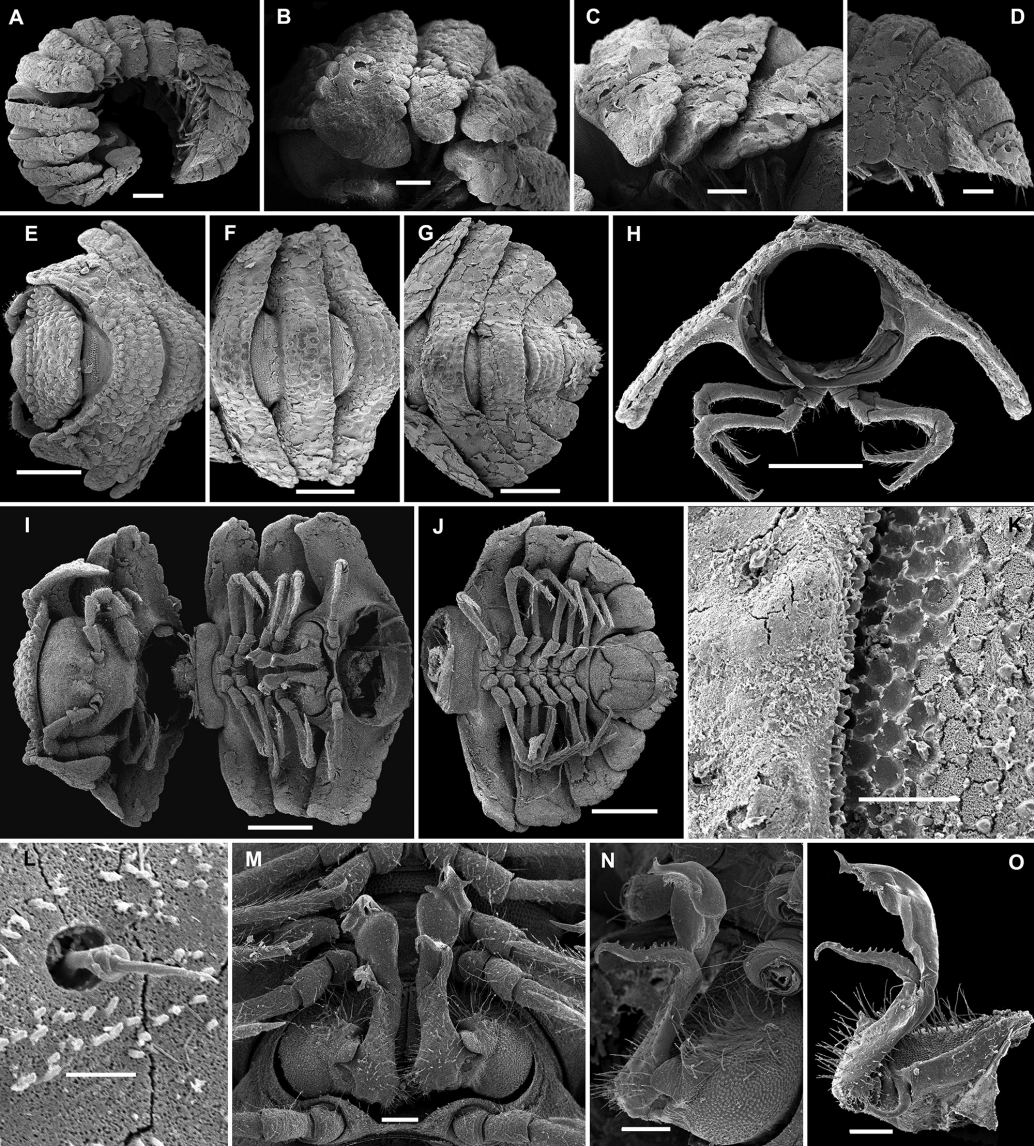
*Eutrichodesmus trontelji* sp. n., ♂ non-type from La Tai Dong; **A** habitus, lateral view **B, E, I** anterior part of body, lateral, dorsal and ventral views, respectively **C, F** midbody segments, lateral and dorsal views, respectively **D, G, J** posterior part of body, lateral, dorsal and ventral views, respectively **H** cross-section of a midbody segment, caudal view **K** limbus, prozonite texture and tergal setae, dorsal view **L** tergal seta, subdorsal view **M** both gonopods in situ, ventral view **N** left gonopod, lateral view **O** right gonopod, mesal view. Scale bars: 0.5 mm (**A, E–J**), 0.2 mm (**B–D**), 0.1 mm (**M–O**), 0.05 mm (**K**), 0.01 mm (**L**).

Figs 7 , 8 , 9 , 10 , 11

#### Type material.

Holotype ♂ (MNHN JC 370), China, Guizhou Prov., Libo County, Libo, Cave Feng Dong, 07.III.1995, leg. P. Trontelj.

Paratypes: 1 ♂, 1 juv. ♀ (MNHN JC 370), 1 ♂ (SEM), same data, together with holotype.

Non-types: 1 ♂, 2 ♀, 11 subadult ♀ or juv. (MNHN JC 370), 1 ♂ (SEM), China, Guizhou Prov., Libo County, Shuipa, Cave Shui Jiang Dong – Cave Shuipu Da Dong, 28.II.1995, leg. P. Trontelj; 1 ♂ (MNHN JC 370), 1 ♂ (SEM), Guizhou Prov., Libo County, Jia Ban, Cave La Tai Dong, 06.III.1995, leg. P. Trontelj.

#### Name.

In honour of Peter Trontelj, the collector.

#### Diagnosis.

Differs from congeners by the particularly broad and moderately declivous paraterga which are set at about 45° to the vertical axis and continue the outline of the sides above paraterga, coupled with mostly 4-5 irregular rows of flat setigerous tubercles/bosses per metatergum, the calyx-shaped tergal setae, and the fairly complex gonopod (see also Key below).

#### Description.

Length of adults ca 8-9 mm, width 1.2 and 2.2 mm (♂ paratype and one ♀ non-type from Shui Jiang Dong) to 1.5 and 2.5 mm (♂ holotype and other non-types) on midbody pro- and metazonae, respectively. Coloration entirely pallid, except some traces of reddish earth material on paraterga.

All characters as in *Eutrichodesmus triangularis* sp. n., except as follows.

Body with 20 segments (♂, ♀), conglobation pattern typical of “doratodesmoids”, volvation apparently being incomplete because of particularly broad and only moderately declivous paraterga. Antennae rather long and poorly clavate (Figs 7I, 9H, 11I). Collum not covering the head from above, fore margin clearly lobulate and slightly elevated, with abundant flat tubercles/bosses arranged in regular rows only at anterior and posterior margins. Metaterga behind collum with three transverse, rather irregular, mixostictic rows of similarly evident, rounded, setigerous tuberculations extending onto paraterga, usually about 10-11+10-11 per row (Figs 7A–G, 9A–F, 11A–G); mid-dorsal regions of metaterga not elevated; caudomarginal lobulations numerous, usually evident across the dorsum (Figs 7A–D, 9A–C, 11A–D); limbus microcrenulate (Figs 7K, 9N, 11K). Paraterga with evident shoulders anteriorly, very broad, moderately declivous, directed ventrolaterad at about 45° to similarly declined sides above paraterga (Figs 7H, 9K, 11H), tips lying very clearly below level of venter, usually rather vaguely uni- to quadrilobate laterally, gradually increasing in number towards paraterga 19; anterolaterals usually wanting, but evident on segment 2 (Figs 7A-D, 9A-C, 11A-D). Paraterga 2 strongly enlarged, directed ventrad (Figs 7A, B, E, I, 9A, D, H, 11A, B, E, I), lateral margin broadly rounded, with few, but evident lobulations; a full row of caudolaterals located above schism, both schism and hyposchism being small (Figs 7B, 9A, 11B). Tergal setae short, 2-segmented, calyx-shaped, apical part setoid (Figs 7L, M, 9L–O, 11L). Pore formula normal, ozopores indistinct, located at about halfway of paratergite and well removed from lateral margin. Epiproct strongly flattened dorsoventrally, densely tuberculate (Figs 7A, D, G, J, 9C, F, J, 11A, D, G, J). Hypoproct subtrapeziform (Figs 7J, 9J, 11J).

Sterna usually with a rather deep, narrow depression between coxae (Figs 7I, J, 9I, 11I, J). Legs long and slender, about 1.1–1.2 times as long as body height (Figs 7H, J, 9I, K, 11H–J), only coxae and basal parts of prefemora finely micropapillate (Fig. 8A).

Gonopods (Figs 7N–P, 8B, C, 9Q–S, 10, 11M–O) complex. Coxae subquadrate, large, micropapillate and densely setose on lateral face, with only a small round lobule caudolaterally. Telopodite considerably longer than coxite, moderately curved ventrad, setose over its basal 1/3 until base of a prominent, subspiniform, microtuberculate, sometimes clearly curved, distofemoral process (**dp**), the latter situated at about halfway of telopodite, acropodite twisted, with a longitudinal mesal fold (**fd**) only sometimes extended into an apical tooth (**j**) (non-types), and with (holo- and paratype) or without (non-types) a small ventral tooth (**k**) at about midway; tip acuminate and axe-shaped; seminal groove terminating subapically on an indistinct hairpad.

#### Remarks.

The conspecificity of the non-type samples with *Eutrichodesmus trontelji* sp. n. is documented in Figs 9–11. It is also corroborated by provenance from the same karst in Libo County, Guizhou Province. Small variations seem to only concern gonopod structure, i.e. the presence in the gonopods of the types of a small tooth **k** and the absence of a tooth **j**.

Interestingly, calyx-shaped tergal setae among *Eutrichodesmus* are also observed only in two cavernicolous species from Guangxi: *Eutrichodesmus latus* and *Eutrichodesmus similis* (see Golovatch et al. 2009a).

### Eutrichodesmus latellai

Taxon classificationAnimaliaPolydesmidaHaplodesmidae

Golovatch, Geoffroy, Mauriès & VandenSpiegel sp. n.

http://zoobank.org/301C4BE2-3354-44DD-95CA-E58E7C830236

Figs 12 , 13

#### Type material.

Holotype ♂ (MNHN JC 371), China, Guizhou Prov., Zhen Feng County, Bei Pan Jiang Town, Cave Shui Chi Dong (Water Pool Cave), ca. 1060 m a.s.l., 31.VII.2005, leg. L. Latella & D. Avesani.

Paratypes: 1 ♂, 2 ♀ (MNHN JC 371), 1 ♀ (SEM), same data, together with holotype.

#### Name.

In honour of Leonardo Latella, one of the main collectors.

#### Diagnosis.

Differs from congeners by the broad and moderately declivous paraterga which are set at about 45° to the vertical axis and almost continue the outline of the sides above paraterga, coupled with three irregular rows of flat setigerous bosses per metatergum, and the especially simple gonopod (see also Key below).

#### Description.

Length of adults ca 12–13 mm, width 1.1–1.2 and 2.8–3.0 mm on midbody pro- and metazonae, respectively (♂, ♀). Holotype ca 12 mm long, 1.2 and 3.0 mm wide on midbody pro- and metazonae, respectively. Coloration entirely pallid, except some traces of reddish earth material on terga.

All characters as in *Eutrichodesmus triangularis* sp. n., except as follows.

**Figure 12.**
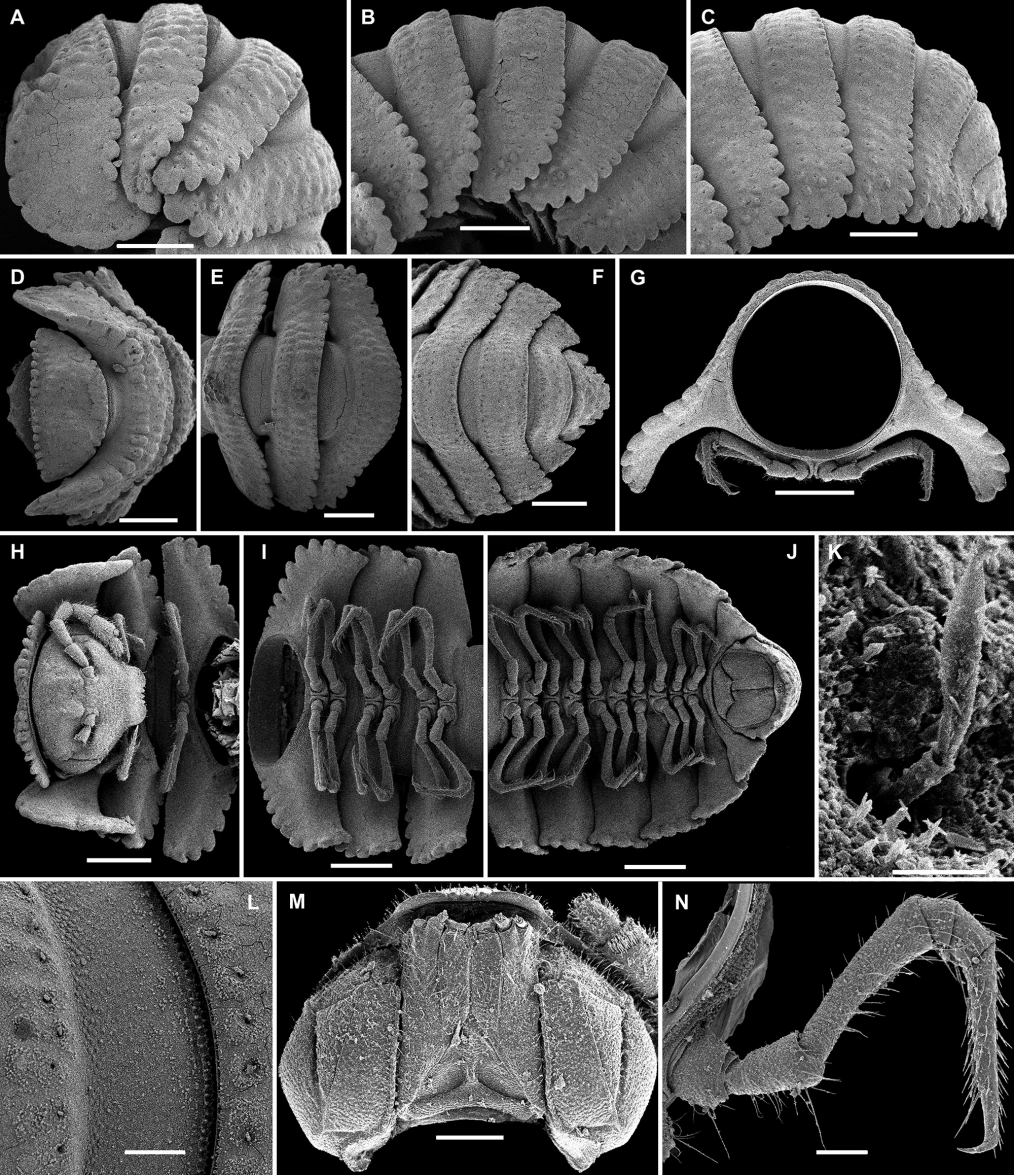
*Eutrichodesmus latellai* sp. n., ♀ paratype; **A, D, H** anterior part of body, lateral, dorsal and ventral views, respectively **B, E, I** midbody segments, lateral, dorsal and ventral views, respectively **C, F, J** posterior part of body, lateral, dorsal and ventral views, respectively **G** cross-section of a midbody segment, caudal view **K** tergal seta, subdorsal view **L** limbus, prozonite texture and tergal setae, dorsal views **M** head, ventral view **N** midbody leg, lateral view. Scale bars: 0.5 mm (**A–J**), 0.2 mm (**M**), 0.1 mm (**L, N**), 0.01 mm (**K**).

Body with 20 segments (♂, ♀), conglobation pattern typical of “doratodesmoids”, volvation apparently being incomplete because of particularly broad and only moderately declivous paraterga. Antennae rather long and poorly clavate (Fig. 12H). Collum not covering the head from above, fore margin clearly lobulate and slightly elevated, with abundant flat bosses arranged in regular rows only at anterior and posterior margins. Metaterga behind collum with three transverse, rather irregular, mixostictic rows of similarly flat, often obliterate and longitudinally oblong, setigerous bosses extending onto paraterga, usually about 15-16+15-16 per row (Fig. 12A–F); mid-dorsal regions of metaterga not elevated; caudomarginal lobulations numerous, usually evident across the dorsum (Fig. 12A–F, H, I); limbus microcrenulate (Fig. 12L). Paraterga with evident shoulders anteriorly, very broad, moderately declivous, directed ventrolaterad at about 45° to similarly declined sides above paraterga (Fig. 12A–G), tips lying clearly below level of venter, usually rather distinctly tri- to quadrilobate laterally, gradually increasing in number towards paraterga 19; anterolaterals usually wanting, but very evident on segment 2 (Fig. 12A, D). Paraterga 2 strongly enlarged, directed ventrad (Fig. 12A, D, H), lateral margin broadly rounded, with few, but very evident lobulations; a full row of caudolaterals located above schism, both schism and hyposchism being small (Fig. 12A). Tergal setae short, 2-segmented, apical part setoid (Fig. 12K). Pore formula normal, ozopores indistinct, located on top of small knobs at about middle of paratergite and well removed from lateral margin (Fig. 12A–C). Epiproct strongly flattened dorsoventrally (Fig. 12C, F, J). Hypoproct subtrapeziform (Fig. 12J).

**Figure 13.**
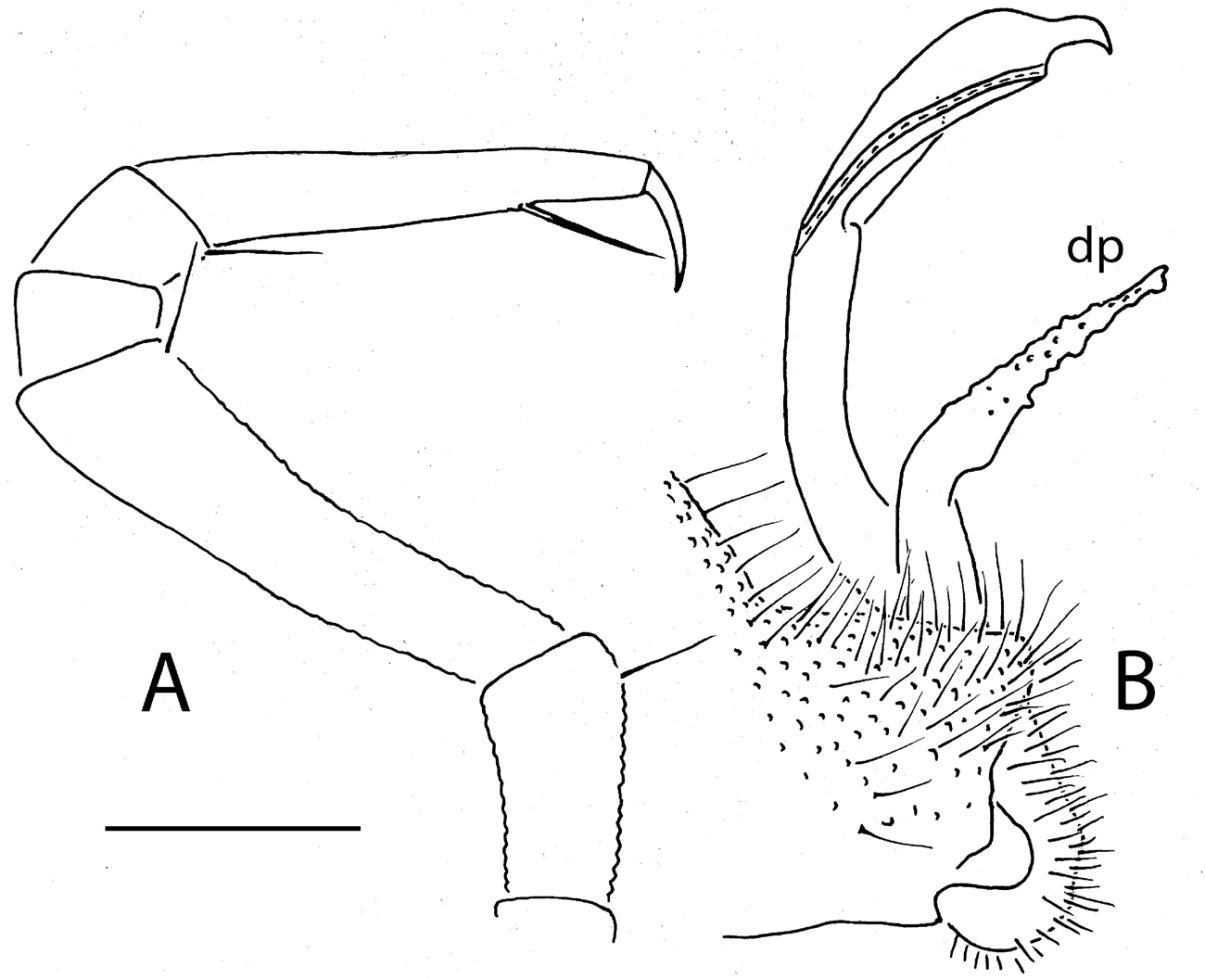
*Eutrichodesmus latellai* sp. n., ♂ paratype; **A** leg 9, lateral view **B** right gonopod, lateral view. Scale bar: 0.2 mm. Designation in text.

Sterna usually with a rather deep, narrow depression between coxae (Fig. 12I, J). Legs long and slender, about as long as body height (Fig. 12G–J), only coxae and most surface of of prefemora finely micropapillate (Figs 12N, 13A).

Gonopods (Fig. 13B) simple. Coxae subquadrate, large, micropapillate and densely setose on lateral face, with only a small round lobule caudolaterally. Telopodite considerably longer than coxite, moderately curved ventrad, setose over its basal 1/3 until base of a prominent, subspiniform, microtuberculate, distofemoral process (**dp**), the latter situated at about basal 1/3 of telopodite, acropodite twisted, devoid of any outgrowths; tip acuminate and beak-shaped; seminal groove terminating subapically; a hairpad wanting.

#### Remark.

More information on this cave and its fauna can be found in Latella and Hu (2008) and in Latella and Zorzin (2008).

### Eutrichodesmus obliteratus

Taxon classificationAnimaliaPolydesmidaHaplodesmidae

Golovatch, Geoffroy, Mauriès & VandenSpiegel sp. n.

http://zoobank.org/41C92D05-2DF5-4365-8F2D-170EA2D3EB5D

Figs 14 , 15

#### Type material.

Holotype ♂ (MNHN JC 372), China, Guizhou Prov., Guanling County, Huajiang Town, Cave Huashiban Dong (Slippery Cave), 26.VII.2005, leg. L. Latella & D. Avesani.

Paratypes: 1 ♂ (MNHN JC 372), 1 ♀ (SEM), same data, together with holotype.

#### Name.

To emphasize the mostly obliterate metatergal tuberculation; adjective.

#### Diagnosis.

Differs from congeners by the largely obliterate metatergal tuberculation (even those at the fore margin of metetergum 2), the rather broad and strongly declivous paraterga which are set at about 30° to the vertical axis and continue the outline of the sides above paraterga, coupled with three irregular rows of very flat setigerous bosses per metatergum, and the fairly complex gonopod telopodite (see also Key below).

#### Description.

Length of adults ca 10 mm, width 1.0–1.1 and 2.1–2.2 mm on midbody pro- and metazonae, respectively (♂, ♀). Holotype ca 1.1 and 2.2 mm wide on midbody pro- and metazonae, respectively. Coloration entirely pallid.

All characters as in *Eutrichodesmus triangularis* sp. n., except as follows.

**Figure 14.**
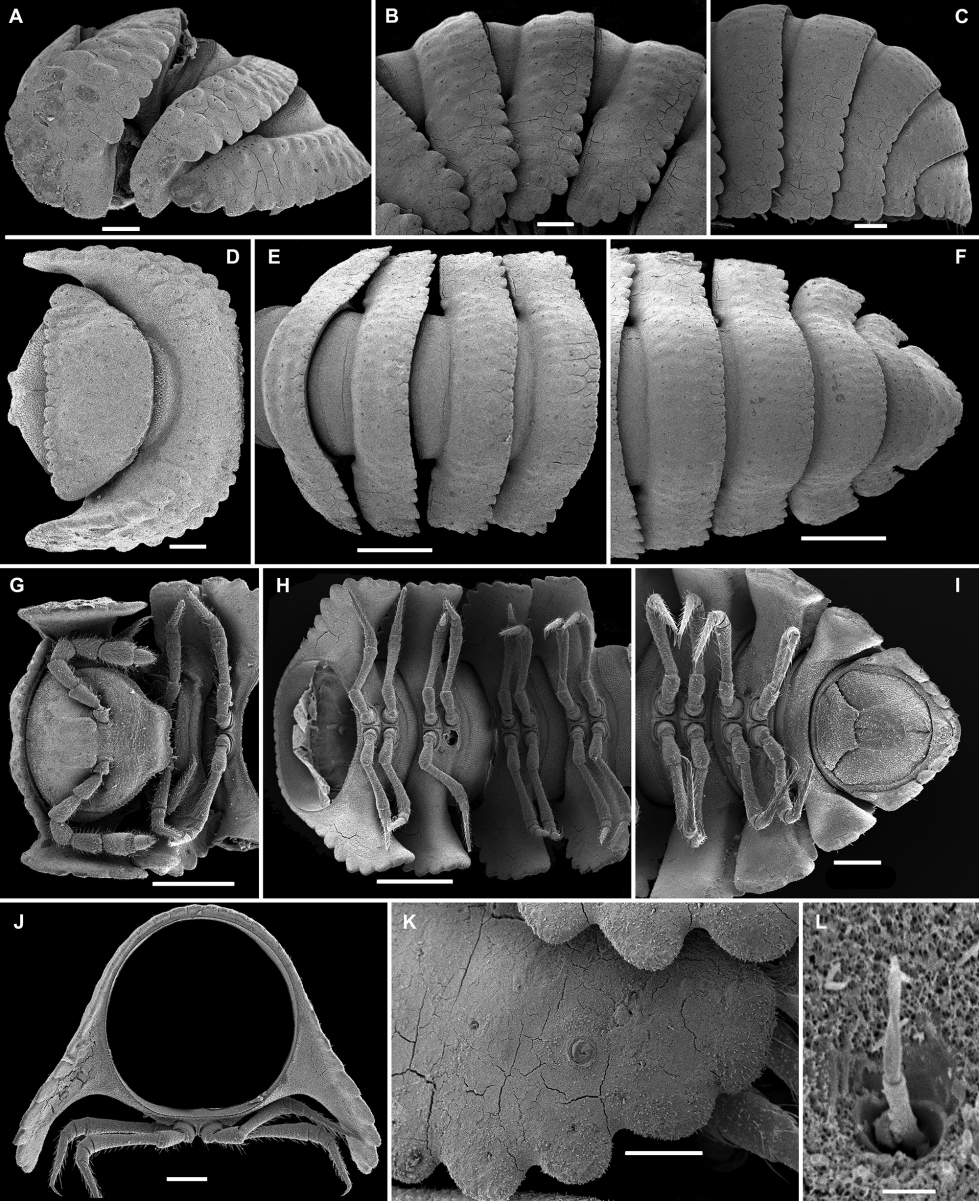
*Eutrichodesmus obliteratus* sp. n., ♀ paratype; **A, D, G** anterior part of body, lateral, dorsal and ventral views, respectively **B, E, H** midbody segments, lateral, dorsal and ventral views, respectively **C, F, I** posterior part of body, lateral, dorsal and ventral views, respectively **J** cross-section of a midbody segment, caudal view **K** paratergite with ozopore, lateral view **L** tergal seta, subdorsal view. Scale bars: 0.5 mm (**E–H**), 0.2 mm (**A–D, I, J**), 0.1 mm (**K**), 0.005 mm (**L**).

Body with 20 segments (♂, ♀), conglobation pattern typical of “doratodesmoids”, volvation apparently being incomplete because of broad and only rather strongly declivous paraterga. Antennae rather long and poorly clavate (Fig. 14G). Collum not covering the head from above, fore margin clearly lobulate and slightly elevated, with abundant, flat, mostly obliterate bosses arranged in a regular row of lobulations only at anterior margin (Fig. 14A, D, G). Metaterga behind collum with three transverse, rather irregular, mixostictic rows of similarly flat, largely obliterate, longitudinally oblong, setigerous bosses extending onto paraterga, usually about 13-14+13-14 per row (Fig. 14A–F); mid-dorsal regions of metaterga not elevated; caudomarginal lobulations numerous, usually evident across the dorsum (Fig. 14A–F); limbus microcrenulate. Paraterga with evident shoulders anteriorly, very broad, rather strongly declivous, directed ventrolaterad at about 70° to similarly declined sides above paraterga (Fig. 14J), tips lying clearly below level of venter, usually rather distinctly tri- to quadrilobate laterally, gradually increasing in number towards paraterga 19; anterolaterals usually wanting, even on segment 2 rather vague (Fig. 14A, G). Paraterga 2 strongly enlarged, directed ventrad (Fig. 14A, D, H), lateral margin broadly rounded, with few, rather vague lobulations; a full row of caudolaterals located above schism, both schism and hyposchism being small (Fig. 14A). Tergal setae short, 2-segmented, apical part setoid (Fig. 14L). Pore formula normal, ozopores indistinct, open flush on surface and located at about caudal 1/3 of paratergite and well removed from lateral margin (Fig. 14K). Epiproct strongly flattened dorsoventrally (Fig. 14C, F, I). Hypoproct subtrapeziform (Fig. 14I).

**Figure 15.**
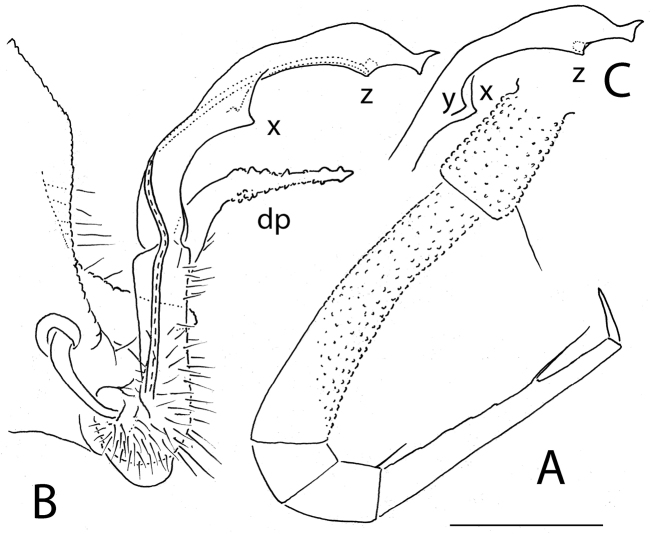
*Eutrichodesmus obliteratus* sp. n., ♂ paratype; **A** leg 9, lateral view **B** right gonopod, mesal view **C** mirrored distal half of right gonopod, lateral view. Scale bar: 0.2 mm. Designations in text.

Sterna usually with a rather deep, narrow depression between coxae (Fig. 14G–I). Legs long and slender, about as long as body height (Figs 14G–J, 15A), only coxae and most surface of prefemora finely micropapillate (Fig. 15A).

Gonopods (Fig. 15B, C) rather complex. Coxae subquadrate, large, micropapillate and densely setose on lateral face, with only a small round lobe caudolaterally. Telopodite considerably longer than coxite, moderately curved ventrad, setose over its basal 1/3 until base of a prominent, subspiniform, abundantly microtuberculate, distofemoral process (**dp**), the latter situated at about basal 1/3 of telopodite, acropodite twisted, in basal 1/3 with two small, flat, subtriangular teeth, one, larger, mesal (**x**), the other, smaller, lateral (**y**); tip acuminate and axe-shaped; seminal groove terminating subapically on another low, subtriangular tooth (**z**); a hairpad wanting.

#### Remark.

More information on this cave and its fauna can be found in Latella and Hu (2008) and in Latella and Zorzin (2008).

### Eutrichodesmus sketi

Taxon classificationAnimaliaPolydesmidaHaplodesmidae

Golovatch, Geoffroy, Mauriès & VandenSpiegel sp. n.

http://zoobank.org/2CA87D80-05A2-4BF9-A70E-1DAB4C79E1A7

Figs 16 , 17

#### Type material.

Holotype ♂ (MNHN JC 373), China, Hunan Prov., Longshan County, Huaoyan, Cave Feihu Dong (33a), 13.IV.1997, leg. B. Sket, Cao & R. Verovnik.

Paratype: 1 subadult ♀ (SEM), same data, together with holotype.

#### Name.

In honour of Boris Sket, one of the main collectors.

#### Diagnosis.

Differs from congeners by the relatively narrow and strongly declivous paraterga which are set low on the body at about 30° to the vertical axis and nearly continue the outline of the sides above paraterga, the low, but distinct, rounded, metatergal bosses arranged in three transverse irregular rows, and the rather simple gonopod (see also Key below).

#### Description.

Length of holotype ca 7 mm, width 1.0 and 1.7 mm on midbody pro- and metazonae, respectively. Coloration entirely pallid.

All characters as in *Eutrichodesmus triangularis* sp. n., except as follows.

**Figure 16.**
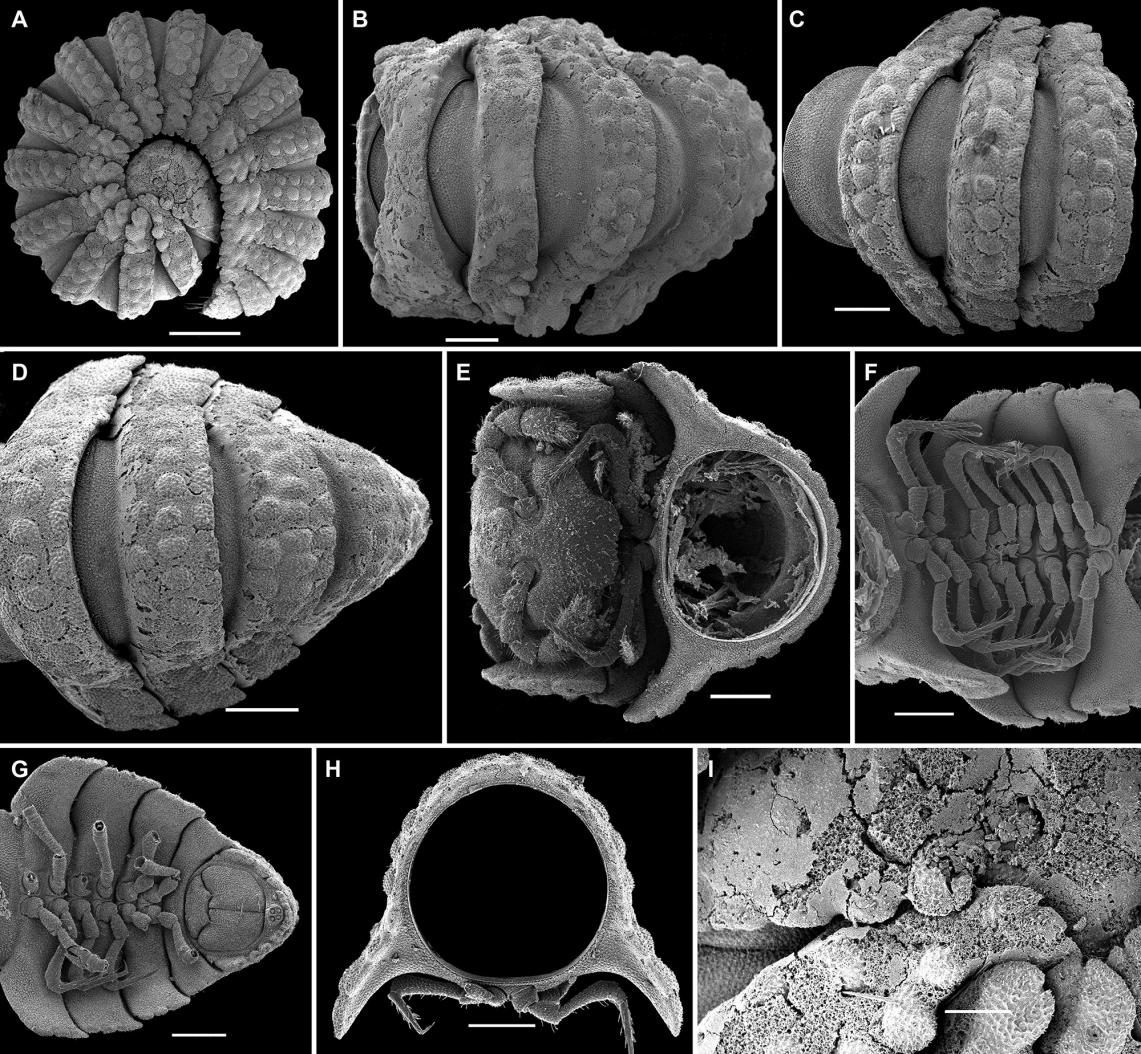
*Eutrichodesmus sketi* sp. n., subadult ♀ paratype; **A** habitus, lateral view **B, E** anterior part of body, dorsal and ventral views, respectively **C, F** midbody segments, dorsal and ventral views, respectively **D, G** posterior part of body, dorsal and ventral views, respectively **H** cross-section of a midbody segment, caudal view **I** schism and hyposchism region, lateral view. Scale bars: 0.5 mm (**A**), 0.2 mm (**B–H**), 0.1 mm (**I**).

Body with 20 segments (♂), conglobation pattern typical of “doratodesmoids”, volvation apparently being complete because of narrow and strongly declivous paraterga. Antennae rather short and clavate (Fig. 16E). Collum not covering the head from above, fore margin clearly lobulate and slightly elevated, with abundant, flat, mostly obliterate bosses arranged in a regular row of lobulations only at anterior margin. Metaterga behind collum with three transverse, rather irregular, mixostictic rows of similarly flat, but rather distinct, rounded, setigerous bosses extending onto paraterga, usually about 9-10+9-10 per row (Fig. 16A); mid-dorsal regions of metaterga not elevated; caudomarginal lobulations numerous, usually evident across the dorsum (Fig. 16A–D); limbus microcrenulate. Paraterga with evident shoulders anteriorly, rather narrow, strongly declivous, directed ventrolaterad at about 70° to even more strongly declined sides above paraterga (Fig. 16E, H), tips lying clearly below level of venter, usually distinctly trilobate laterally; anterolaterals evident only in segment 2 (Fig. 16A). Paraterga 2 strongly enlarged, directed ventrad (Fig. 16A, E), lateral margin broadly rounded, with few, rather distinct lobulations; a full row of caudolaterals located above schism, both schism and hyposchism being small (Fig. 16A, I). Tergal setae short, 2-segmented, apical part setoid (Fig. 16I). Pore formula normal, ozopores indistinct, open flush on surface and located at about caudal 1/3 of paratergite above caudal lobulation and well removed from lateral margin (Fig. 16A, I). Epiproct strongly flattened dorsoventrally (Fig. 16D, G). Hypoproct subtrapeziform (Fig. 16G).

**Figure 17.**
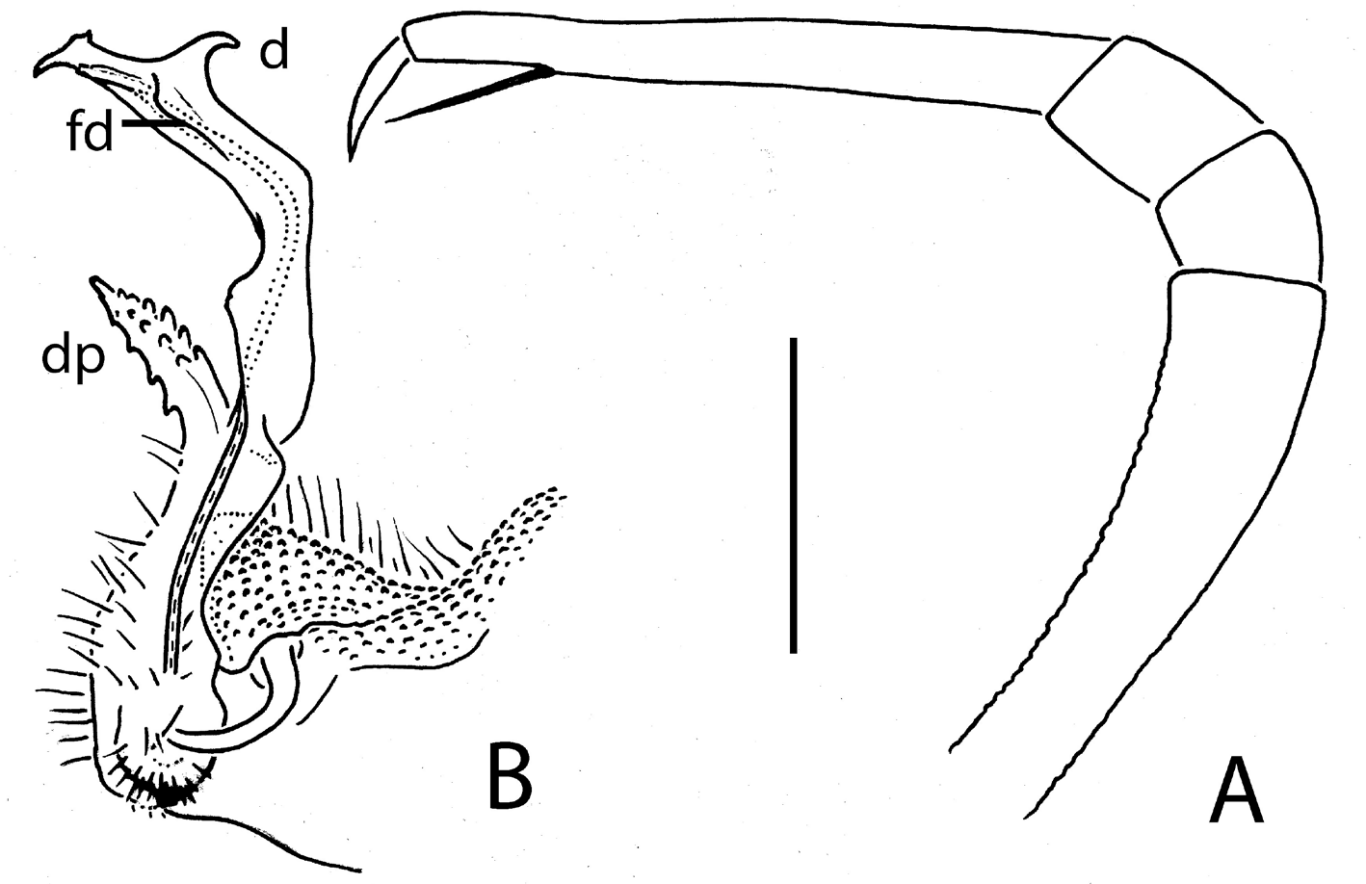
*Eutrichodesmus sketi* sp. n., ♂ holotype; **A** leg 9, lateral view **B** right gonopod, mesal view. Scale bar: 0.2 mm. Designations in text.

Sterna usually with a rather deep, narrow depression between coxae (Fig. 16G–I). Legs long and slender, about as long as body height (Fig. 16F, G), only coxae and most surface of prefemora finely micropapillate (Fig. 17A).

Gonopods (Fig. 17B) rather simple. Coxae subquadrate, large, micropapillate and densely setose mostly on lateral face, with only a small round lobe caudolaterally. Telopodite considerably longer than coxite, moderately curved ventrad, setose over its basal 1/3 until base of a prominent, subspiniform, stout, abundantly microtuberculate, distofemoral process (**dp**), the latter situated at about basal 1/3 of telopodite, acropodite twisted, distal 1/3 with a small mesal fold (**fd**) and a strong, recurved, ventral tooth (**d**); tip acuminate and axe-shaped; seminal groove terminating subapically; a hairpad wanting.

### Eutrichodesmus apicalis

Taxon classificationAnimaliaPolydesmidaHaplodesmidae

Golovatch, Geoffroy, Mauriès & VandenSpiegel sp. n.

http://zoobank.org/3A82B084-C569-477F-AF3A-C305E8C37745

Figs 18 , 19

#### Type material.

Holotype ♂ (MNHN JC 374), China, Hubei Prov., Yishang Yichang County, Grotte des Araignées, 15.VIII.1992, leg. J. Lips (K1-2).

Paratype: 1 ♂ (SEM), same data, together with holotype.

#### Name.

To emphasize the apical termination of the seminal groove; adjective.

#### Diagnosis.

Differs from congeners by the relatively narrow and strongly declivous paraterga which are set low on the body at about 40° to the vertical axis and distinctly discontinue the subvertical outline of the sides above paraterga, coupled with narrow paraterga which only slightly overreach the level of the venter, the low, but distinct, rounded, metatergal tuberculations arranged in three transverse irregular rows, and the rather complex gonopod (see also Key below).

#### Description.

Length of holotype ca 7 mm, width 0.6 and 1.0 mm on midbody pro- and metazonae, respectively. Coloration entirely pallid.

All characters as in *Eutrichodesmus triangularis* sp. n., except as follows.

**Figure 18.**
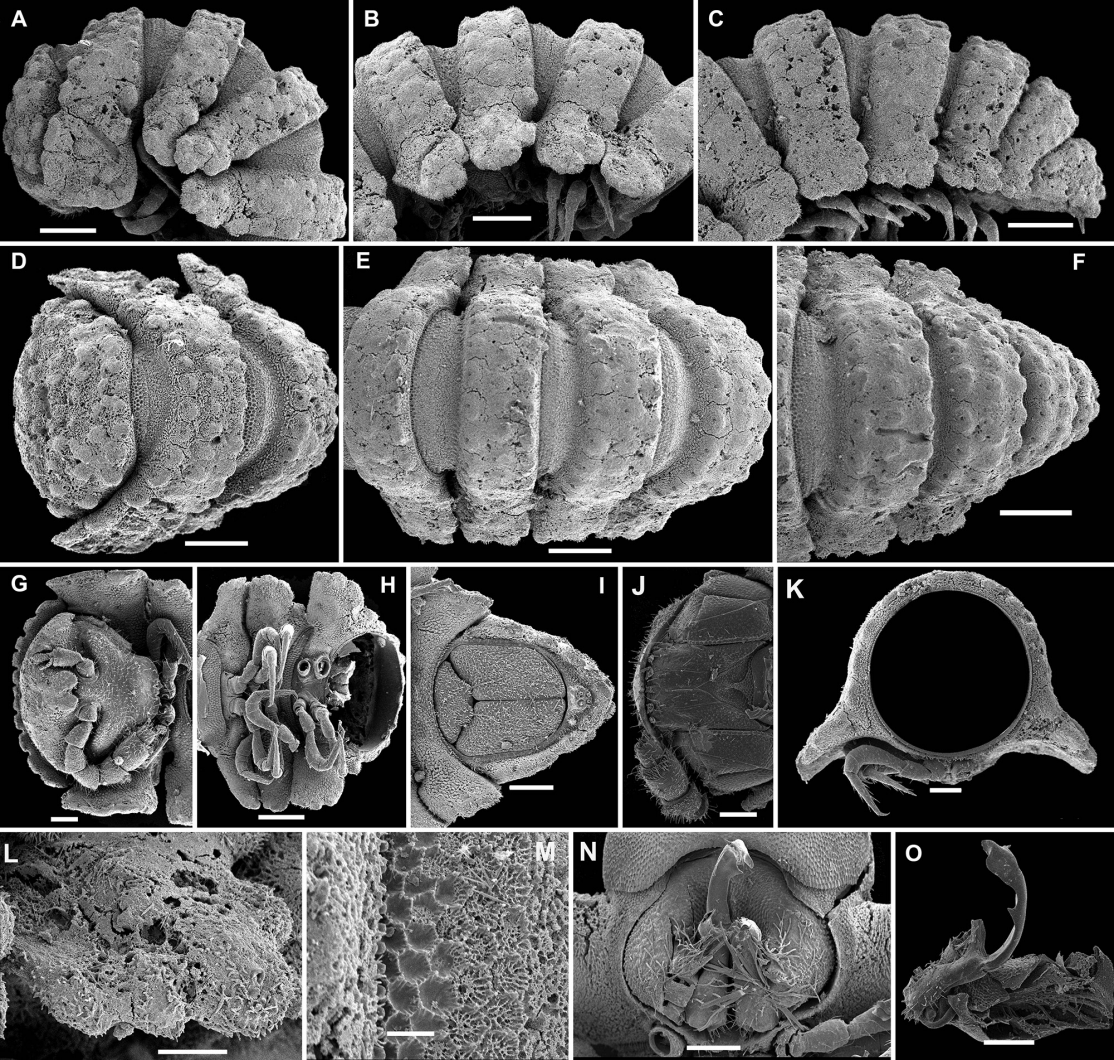
*Eutrichodesmus apicalis* sp. n., ♂ paratype; **A, D, G** anterior part of body, lateral, dorsal and ventral views, respectively **B, E, H** midbody segments, lateral, dorsal and ventral views, respectively **C, F, I** posterior part of body, lateral, dorsal and ventral views, respectively **J** head, ventral view **K** cross-section of a midbody segment, caudal view **L** midbody paratergite, lateral view **M** limbus and prozonite texture, dorsal view **N** both gonopods in situ, ventral view **O** right gonopod, mesal view. Scale bars: 0.2 mm (**A–F, H**), 0.1 mm (**G, I–K, N, O**), 0.05 mm (**L**), 0.02 mm (**M**).

Body with 20 segments (♂), conglobation pattern typical of “doratodesmoids”, volvation apparently being complete because of narrow and strongly declivous paraterga. Antennae rather short and clavate (Fig. 18G, J). Collum not covering the head from above, fore margin clearly lobulate and slightly elevated, with abundant, mostly distinct bosses or tuberculations arranged in regular rows of lobulations only at anterior and posterior margins. Metaterga behind collum with three transverse, rather irregular, mixostictic rows of similarly distinct, rounded, setigerous tuberculations extending onto paraterga, usually about 6-7+6-7 per row (Fig. 18A–F); mid-dorsal regions of metaterga not elevated; caudomarginal lobulations few, usually evident only near bases of paraterga (Fig. 18A–C); limbus microcrenulate (Fig. 18M). Paraterga with evident shoulders anteriorly, rather narrow, strongly declivous, directed ventrolaterad at about 40° to even more strongly declined, subvertical sides above paraterga (Fig. 18K), tips lying only slightly below level of venter, usually distinctly trilobate laterally; anterolaterals evident only in segment 2 (Fig. 18A, D). Paraterga 2 strongly enlarged, directed ventrad (Fig. 18A, D, G), lateral margin broadly rounded, with few, rather distinct lobulations; a full row of caudolaterals located above schism, both schism and hyposchism being small (Fig. 18A). Tergal setae short, apparently 2-segmented. Pore formula apparently normal, ozopores indistinct. Epiproct strongly flattened dorsoventrally (Fig. 18C, I). Hypoproct subtrapeziform (Fig. 18I).

Sterna usually with a rather deep, narrow depression between coxae (Fig. 18H). Legs rather short, but slender, nearly as long as body height (Fig. 18H, K), only coxae and most surface of prefemora finely micropapillate.

**Figure 19.**
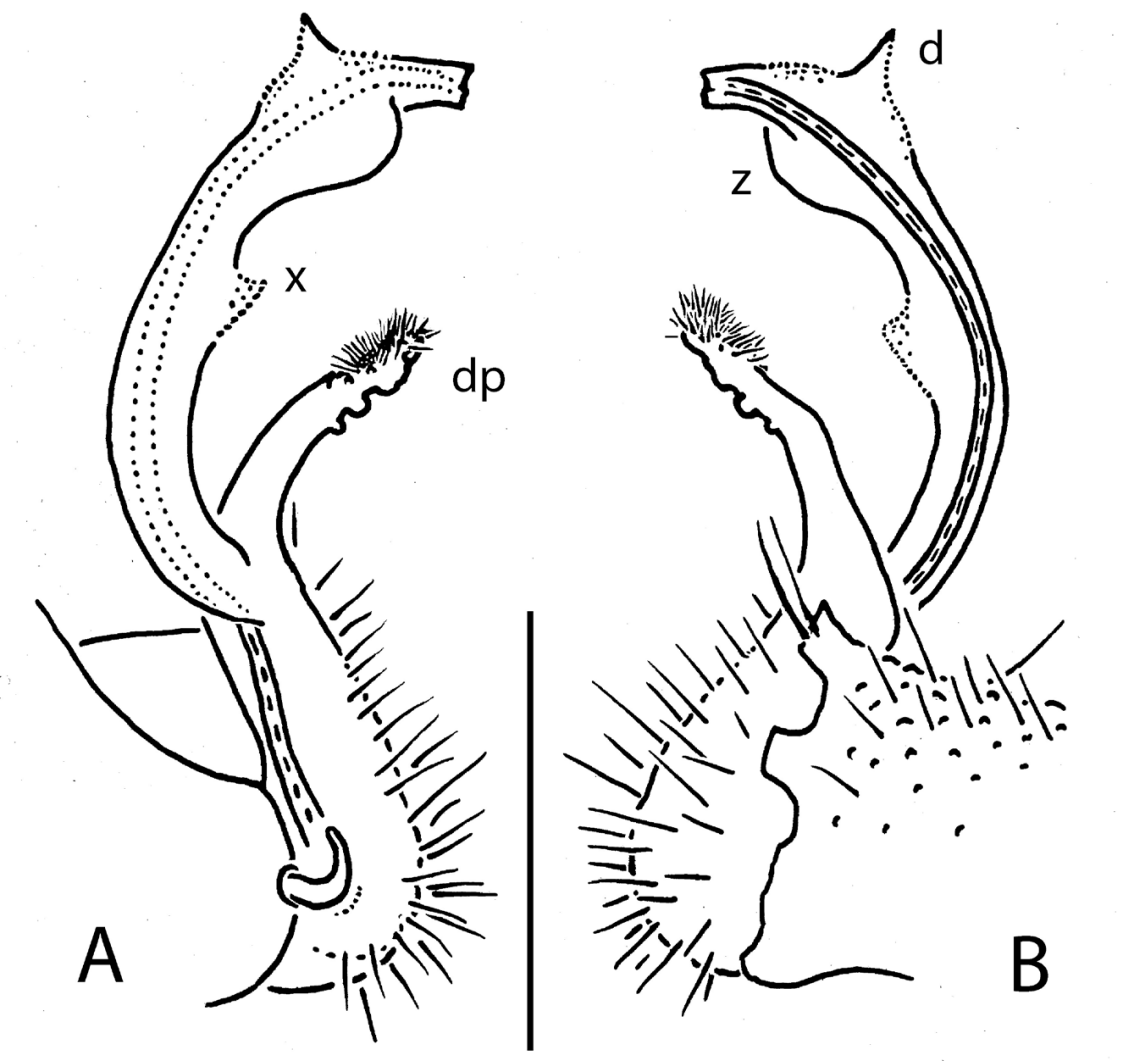
*Eutrichodesmus apicalis* sp. n., ♂ holotype; **A, B** left gonopod, mesal and lateral views, respectively. Scale bar: 0.2 mm. Designations in text.

Gonopods (Figs 18N, O, 19) rather complex. Coxae subquadrate, large, micropapillate and setose on lateral face, with a small, truncate, setigerous tooth caudolaterally. Telopodite considerably longer than coxite, moderately curved ventrad, setose nearly over its basal half until base of a prominent, subspiniform, microtuberculate, subapically micropilose, distofemoral process (**dp**), the latter situated at about basal 1/3 of telopodite, acropodite twisted, with a small, midway, dorsomesal (**x**) and a stronger, subapical, ventral tooth (**d**), the latter located opposite a rounded lobe (**z**); tip subtruncate; seminal groove terminating apically; a hairpad wanting.

#### Remark.

More information on the location of the cave can be found at http://www.groupe-speleo-vulcain.com/explorations/expeditions-a-letranger/

### Eutrichodesmus troglobius

Taxon classificationAnimaliaPolydesmidaHaplodesmidae

Golovatch, Geoffroy, Mauriès & VandenSpiegel sp. n.

http://zoobank.org/BA529FEC-19CB-46E2-867D-B3621667978E

Figs 20 , 21

#### Type material.

Holotype ♂ (MNHN JC 375), China, Guizhou Prov., Kaiyang, Cave Xianyan Dong, 19.II.2004, leg. S. Prevorčnik & B. Sket.

Paratypes: 1 ♂, 2 ♀, 2 subadult ♀ (MNHN JC 375), 1 ♀ (SEM), same data, together with holotype.

#### Name.

To emphasize cavernicoly; adjective.

#### Diagnosis.

Differs from congeners by the relatively broad and modestly declivous paraterga which are set low on the body at about 45° to the vertical axis and distinctly discontinue the more strongly declined outline of the sides above paraterga, coupled with low, but distinct, mostly longitudinally oblong, metatergal tuberculations arranged in three transverse irregular rows, and the rather simple gonopod acropodite which only shows a small distodorsal tooth (see also Key below).

#### Description.

Length of adults ca 8–9 mm, width 1.0 and 1.9–2.0 mm on midbody pro- and metazonae, respectively. Holotype ca 9 mm long, 1.0 and 2.0 mm wide on pro- and metazonae, respectiverly. Coloration entirely pallid.

All characters as in *Eutrichodesmus triangularis* sp. n., except as follows.

**Figure 20.**
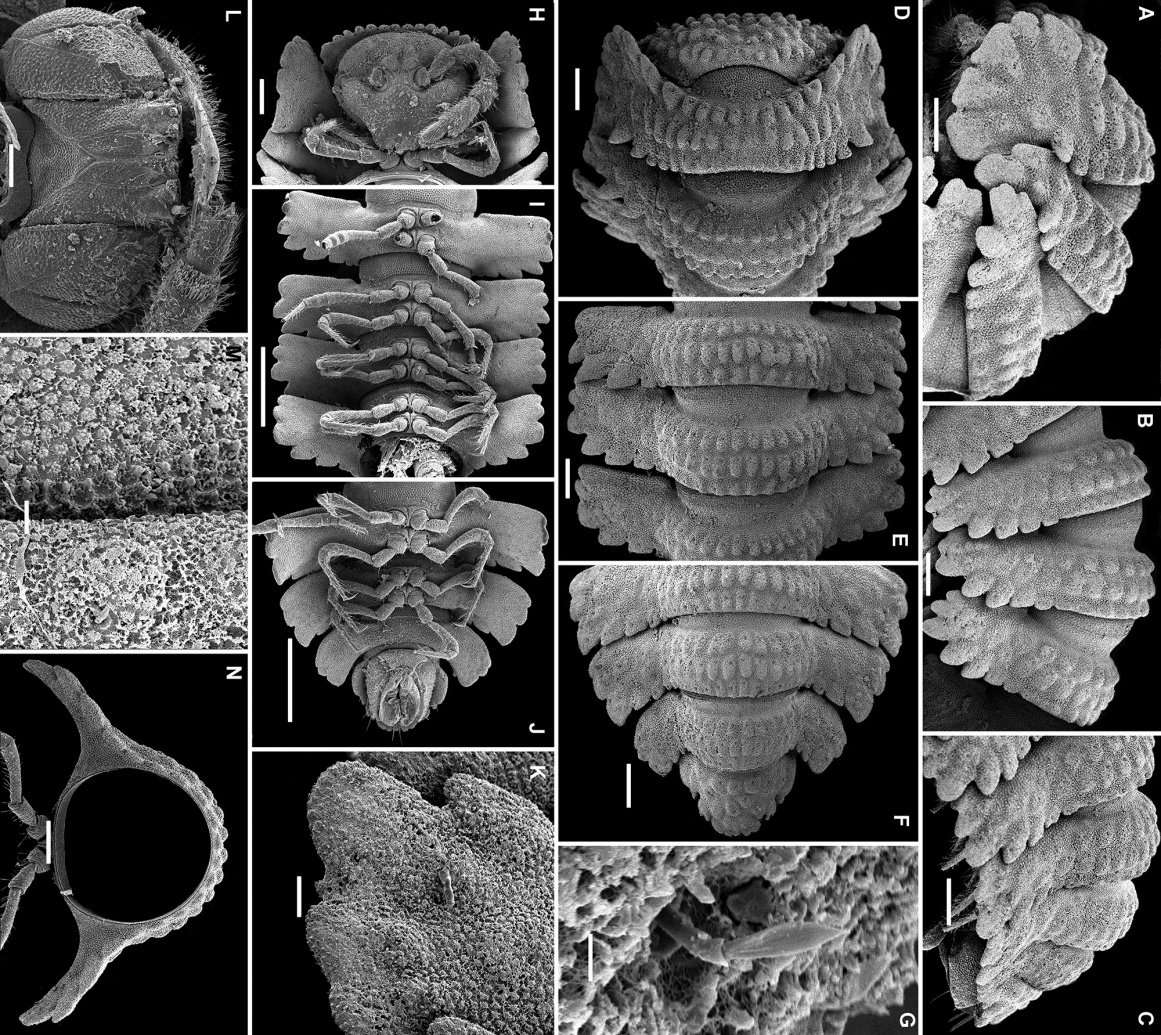
*Eutrichodesmus troglobius* sp. n., ♀ paratype; **A, D, H** anterior part of body, lateral, dorsal and ventral views, respectively **B, E, I** midbody segments, lateral, dorsal and ventral views, respectively **C, F, J** posterior part of body, lateral, dorsal and ventral views, respectively **G** tergal seta, subdorsal view **K** midbody paratergite, lateral view **L** head, ventral view **M** limbus and prozonite texture, dorsal view **N** cross-section of a midbody segment, caudal view. Scale bars: 0.5 mm (**I, J**), 0.2 mm (**A–F, H, N**), 0.1 mm (**L**), 0.02 mm (**M**).

Body with 20 segments (♂, ♀), conglobation pattern typical of “doratodesmoids”, volvation apparently being incomplete because of broad and modestly declivous paraterga. Antennae rather long and poorly clavate (Fig. 20H, L). Collum not covering the head from above, fore margin clearly lobulate and slightly elevated, with abundant distinct tuberculations arranged in regular rows, but lobulations observed only at anterior margin. Metaterga behind collum with three transverse, rather irregular, mixostictic rows of similarly distinct, longitudinally oblong, setigerous tuberculations extending onto paraterga, usually about 8-10+8-10 per row (Fig. 20A–F); mid-dorsal regions of metaterga not elevated; caudomarginal lobulations numerous, 2-3 more evident ones only on paraterga (Fig. 20A–F); limbus microcrenulate (Fig. 20M). Paraterga with evident shoulders anteriorly, broad, modestly declivous, directed ventrolaterad at about 45° to even more strongly declined sides above paraterga (Fig. 20N), tips lying clearly below level of venter, usually distinctly trilobate laterally; anterolaterals evident only in segment 2 (Fig. 20A, D). Paraterga 2 strongly enlarged, directed ventrad (Fig. 20A, D, H), lateral margin broadly rounded, with few, but very distinct lobulations; a full row of similarly large caudolaterals located above schism, both schism and hyposchism being small (Fig. 20A). Tergal setae short, 2-segmented, apical part setoid (Fig. 20G). Pore formula apparently normal, ozopores indistinct. Epiproct strongly flattened dorsoventrally and tuberculate dorsally (Fig. 20C, F, J). Hypoproct subtrapeziform (Fig. 20J).

**Figure 21.**
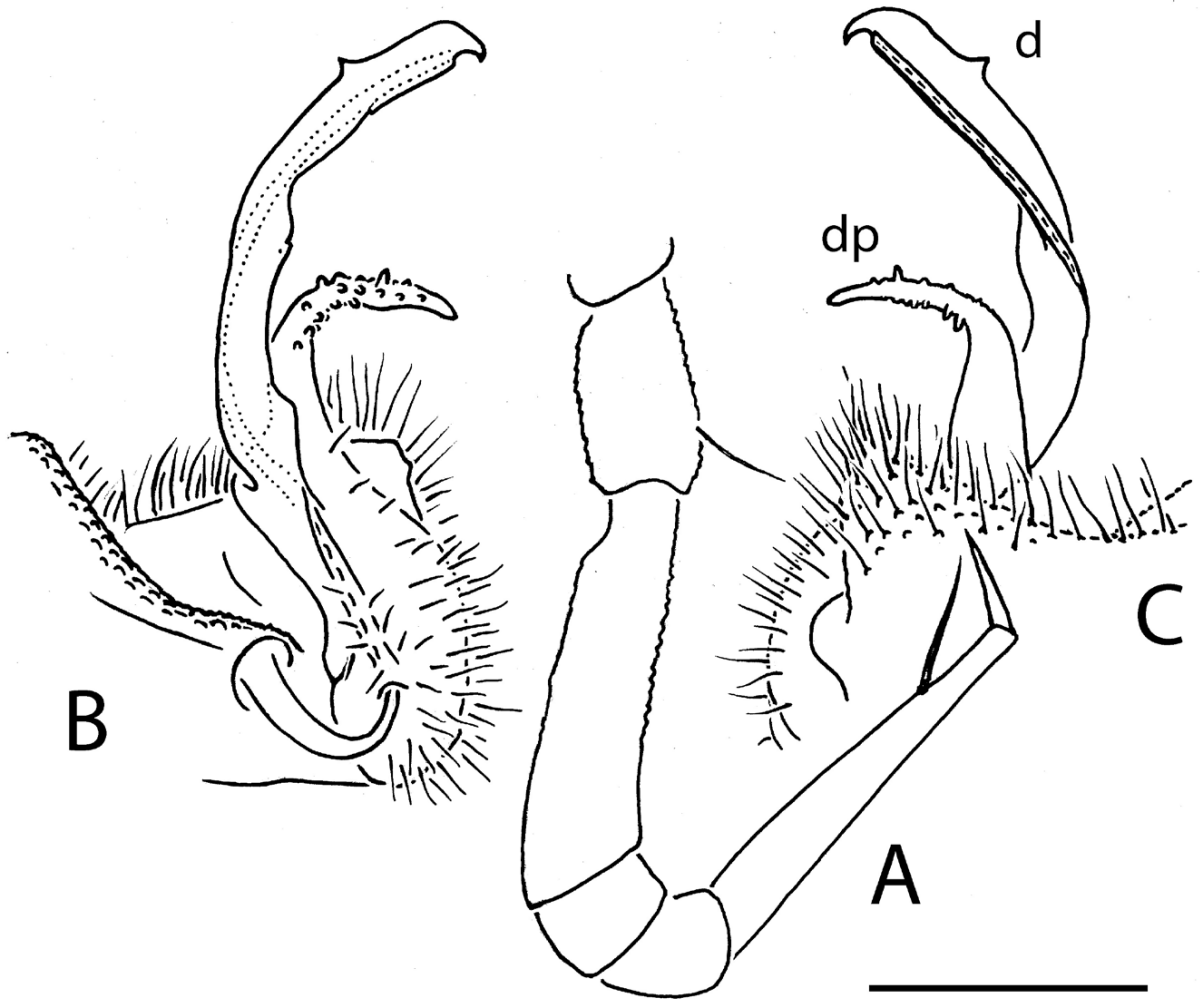
*Eutrichodesmus troglobius* sp. n., ♂ paratype; **A** leg 9, lateral view **B, C** left gonopod, mesal and lateral views, respectively. Scale bar: 0.2 mm. Designations in text.

Sterna usually with a rather deep, narrow depression between coxae (Fig. 20I, J). Legs long and slender, 1.1-1.2 times as long as body height (Fig. 20I, J, N), only coxae and most surface of prefemora finely micropapillate (Fig. 21A).

Gonopods (Fig. 21B, C) rather simple. Coxae subquadrate, large, micropapillate and setose mostly on lateral face, with a small, subtriangular, setigerous tooth caudolaterally. Telopodite considerably longer than coxite, moderately and regularly curved ventrad, setose nearly over its basal half until base of a prominent, curved, subspiniform, abundantly microtuberculate, distofemoral process (**dp**), the latter situated at about basal 1/3 of telopodite, acropodite twisted, with a small, subapical, ventral tooth (**d**); seminal groove terminating subapically; a hairpad wanting.

## Conclusion

The nine new species described here are presumed to be troglobites, as all were collected in caves and all are troglonorphic as evidenced by unpigmented teguments. Their discovery supports the ideas that *Eutrichodesmus* is one of the most speciose millipede genera in China, that the true cavernicoles among *Eutrichodesmus* species are mainly confined to southern China’s karsts, and that many more Chinese *Eutrichodesmus* species are yet to be collected and described.

### Key to *Eutrichodesmus* species currently known to occur in mainland China

**Table:** 

1	Each postcollum metatergum with only two transverse rows of tuberculations or bosses	**the *peculiaris*-group, 2**
–	Each postcollum metatergum with at least three transverse rows of tuberculations or bosses	**4**
2	1+1 mid-dorsal tubercles only slightly higher than others and located only in 2^nd^ row on segments 4-6(7). Zhejiang Province	***Eutrichodesmus pectinatidentis***
–	Mid-dorsal tubercles much higher than others and located in both rows at least on segments 4-16(17)	**3**
3	Most of tuberculations on collum obliterated, retained only near lateral edge. Mid-dorsal tubercles on penultimate segment low, but evident, like a small crest. Chongqing Municipality	***Eutrichodesmus soesilae***
–	Almost entire collum covered with tuberculations. Mid-dorsal tubercles on penultimate segment nearly wanting, flat, not crest-shaped. Fujian Province	***Eutrichodesmus anisodentus***
4	Adult body with 19 segments (Fig. 1). Distofemoral process (**dp**) of gonopod triangular and acuminate (Fig. 2)	***Eutrichodesmus triangularis* sp. n.**
–	Adult body with 20 segments. Distofemoral process of gonopod not triangular and acuminate	**5**
5	At least some metaterga increasingly strongly elevated mid-dorsally towards segment 17 or 18, with 1-2 outgrowths, projections or a ridge (e.g. Figs 3A–H, 5A–G). Three transverse rows of tuberculations or bosses per metatergum	**6**
–	No metaterga elevated mid-dorsally over others. Three or more transverse rows of tuberculations or bosses per metatergum	**10**
6	Mid-dorsal regions of metaterga increasingly strongly elevated towards segment 17 due to enlarged tubercles of middle row, thereafter smaller (Fig. 3A–H). Gonopod distofemoral process (**dp**) held subparallel to acropodite (Figs 3N–Q, 4)	***Eutrichodesmus lipsae* sp. n.**
–	Mid-dorsal regions of metaterga increasingly strongly elevated towards segment 18. Gonopod distofemoral process held subrectangular to acropodite, gap between **dp** and acropodite being considerably wider	**7**
7	Metaterga 7-18 each with an increasingly evident mid-dorsal outgrowth/crest, bimodal due to clearly enlarged 1^st^ and 2^nd^ rows of tuberculations. Distofemoral process of gonopod microtuberculate	***Eutrichodesmus distinctus***
–	Mid-dorsal crests on metaterga unimodal, subtriangular due to only 2^nd^ row of tuberculations being clearly enlarged. Distofemoral process of gonopod a simple long spine	**8**
8	Paraterga narrow, subvertical (Fig. 5L). Gonopod acropodite strongly falcate, seminal groove terminating on a small, but evident hairpad (Fig. 6B). Guizhou	***Eutrichodesmus tenuis* sp. n.**
–	Paraterga considerably broader, directed ventrolaterad. Gonopod acropodite only slightly curved ventrad, devoid of a hairpad. Yunnan	**9**
9	Metatergal tuberculations very small knobs. Gonopod acropodite clearly enlarged relative to distofemoral process, devoid of a distodorsal tooth	***Eutrichodesmus dorsiangulatus***
–	Metatergal tuberculations mostly distinct. Gonopod acropodite slender, with a distodorsal tooth	***Eutrichodesmus monodentus***
10	At least some metaterga with tuberculations/bosses arranged in 4-5 transverse irregular rows	**11**
–	All metaterga with only three transverse rows of tuberculations or bosses	**13**
11	Metaterga 2-13 each with four, following ones with five, rows of tuberculations or bosses,. Guangdong	***Eutrichodesmus digitatus***
–	Metaterga with 3-5 rows of tuberculations or bosses, pattern of increase different	**12**
12	Each postcollum metatergum with 4-5 irregular rows of bosses (Figs 7A–G, 9A–F, 11A–G). Gonopod distofemoral process (**dp**) long and microtuberculate, acropodite with a mesal fold (**fd**), the latter sometimes extended into an apical tooth (**j**) (Figs 8B, C, 10)	***Eutrichodesmus trontelji* sp. n.**
–	Metaterga with 3-4 rows of tuberculations or bosses. Gonopod distofemoral process (**dp**) short and simple, but acropodite enlarged, bipartite and more elaborate	***Eutrichodesmus planatus***
13	Paraterga narrow, set low on body, rather strongly declined ventrolaterad and more or less clearly discontinuing the outline of sides above paraterga	**14**
–	Paraterga broad to very broad, set higher on body to (almost) continue the outline of sides above paraterga	**17**
14	Distofemoral process of gonopod a simple, strong, ventrobasally setose hook directed dorsad. Yunnan	***Eutrichodesmus arcicollaris***
–	Distofemoral process of gonopod more elaborate	**15**
15	Seminal groove terminating apically, distofemoral process (**dp**) of gonopod micropilose apically (Fig. 19). Hubei	***Eutrichodesmus apicalis* sp. n.**
–	Seminal groove terminating subapically, distofemoral process of gonopod devoid of micropilosity	**16**
16	Distofemoral process of gonopod bipartite, long and complex. Guizhou	***Eutrichodesmus incisus***
–	Distofemoral process (**dp**) of gonopod unipartite, short and microtuberculate (Fig. 17B). Hunan	***Eutrichodesmus sketi* sp. n.**
17	Paraterga very broad, each about as wide as prozonite. Guangxi	**18**
–	Paraterga considerably narrower than prozonite width	19
18	Collum devoid of a row of lobulations at fore margin	***Eutrichodesmus latus***
–	Collum with a row of distinct lobulations at fore margin	***Eutrichodesmus similis***
19	Gonopod simple, but unusually strongly falcate. Hunan	***Eutrichodesmus spinatus***
–	Gonopod only slightly to moderately curved	**20**
20	Distofemoral process of gonopod strongly appressed to a simple acropodite. Jiangxi	***Eutrichodesmus simplex***
–	Distofemoral process of gonopod not appressed to often a more elaborate acropodite, gap between both parts being considerable. Guizhou	**21**
21	Gonopod acropodite complex, with a number of teeth (**x**, **y**, **z**), but without distodorsal tooth **d** (Fig. 15B, C)	***Eutrichodesmus obliteratus* sp. n.**
–	Gonopod acropodite simple, at most with a small tooth **d**	**22**
22	Gonopod acropodite with a small tooth **d** (Fig. 21B, C)	***Eutrichodesmus troglobius* sp. n.**
–	Gonopod acropodite devoid of considerable outgrowths (Fig. 13B)	***Eutrichodesmus latellai* sp. n.**

## Supplementary Material

XML Treatment for Eutrichodesmus triangularis

XML Treatment for Eutrichodesmus lipsae

XML Treatment for Eutrichodesmus tenuis

XML Treatment for Eutrichodesmus trontelji

XML Treatment for Eutrichodesmus latellai

XML Treatment for Eutrichodesmus obliteratus

XML Treatment for Eutrichodesmus sketi

XML Treatment for Eutrichodesmus apicalis

XML Treatment for Eutrichodesmus troglobius
